# Supplementary material for: Consensus Ensemble Multitarget Neural Network Model of Anxiolytic Activity of Chemical Compounds and Its Use for Multitarget Pharmacophore Design
Source: Pharmaceuticals (Basel). 2023 May 11;16(5):731. doi: 10.3390/ph16050731 (PMC10223244; doi:10.3390/ph16050731)
Supplement: Supplementary file 1 [file pharmaceuticals-16-00731-s001.zip › TableS1 All92_Targets.pdf]

Table S1. Homo sapiens biotargets relevant to anxiolytic activity and having reliable confirmation in the form of experimental data on the activity of known compounds.

No	Targets	Genes			Main UniProt ID	Main UniProt Name
1	5-ht1	HTR1A			P08908	5-hydroxytryptamine receptor 1A
2	5-hta / 5-ht2c	HTR1A	HTR2C		P08908 / P28335	5-hydroxytryptamine receptor 1A / 5-hydroxytryptamine receptor 2C
3	5-ht4a	HTR4A			Q13639	5-hydroxytryptamine receptor 4A
4	5-ht4b	HTR4B			Q13639	5-hydroxytryptamine receptor 4B
5	5-ht4d	HTR4D			Q13639	5-hydroxytryptamine receptor 4D
6	5-ht4hb	HTR4B			Q13639	5-hydroxytryptamine receptor 4B
7	5-ht7b	HTR7			P34969	5-hydroxytryptamine receptor 7
8	5-ht7l	HTR7			P34969	5-hydroxytryptamine receptor 7
9	5-ht7s	HTR7			P34969	5-hydroxytryptamine receptor 7
10	5-hydroxytryptamine receptor 1a (5-ht1a)	HTR1A			P08908	5-hydroxytryptamine receptor 1A
11	5-hydroxytryptamine receptor 1b	HTR1B			P28222	5-hydroxytryptamine receptor 1B
12	5-hydroxytryptamine receptor 1d	HTR1D			P28221	5-hydroxytryptamine receptor 1D
13	5-hydroxytryptamine receptor 2a (5-ht2a)	HTR2A			P28223	5-hydroxytryptamine receptor 2A
14	5-hydroxytryptamine receptor 2b (5ht2b)	HTR2B			P41595	5-hydroxytryptamine receptor 2B
15	5-hydroxytryptamine receptor 2c (5ht2c)	HTR2C			P28335	5-hydroxytryptamine receptor 2C
16	5-hydroxytryptamine receptor 2c (5-ht2c)	HTR2C			P28335	5-hydroxytryptamine receptor 2C
17	5-hydroxytryptamine receptor 2c	HTR2C			P28335	5-hydroxytryptamine receptor 2C
18	5-hydroxytryptamine receptor 6 (5-ht6)	HTR6			P50406	5-hydroxytryptamine receptor 6
19	5-hydroxytryptamine receptor 7 (5-ht7)	HTR7			P34969	5-hydroxytryptamine receptor 7
20	adrenergic alpha1	ADRA1A			P35348	Alpha-1A adrenergic receptor
21	adrenergic beta-2 receptor (β2)	ADRB2			P07550	Beta-2 adrenergic receptor
22	adrenergic beta-2 receptor (beta2)	ADRB2			P07550	Beta-2 adrenergic receptor
23	adrenergic receptor alpha	ADRA1A	ADRA1B	ADRA1D	P35348 / P35368 / P25100	Alpha-1A / Alpha-1B / Alpha-1D adrenergic receptor
24	adrenergic receptor alpha-1	ADRA1A			P35348	Alpha-1A adrenergic receptor
25	adrenergic receptor beta	ADRB1	ADRB2	ADRB3	P08588 / P07550 / A8KAG8	Beta-1 / Beta-2 / Beta-3 adrenergic receptor
26	adrenergic receptor	—			—	Adrenergic receptor
27	adrenergic receptors; alpha-1 a & b	ADRA1A	ADRA1B		P35348 / P35368	Alpha-1A / Alpha-1B adrenergic receptor
28	adrenergic, alpha-2b-, receptor [homo sapiens]	ADRA2B			P18089	Alpha-2B adrenergic receptor
29	aldehyde dehydrogenase (aldh2)	ALDH2			P05091	Aldehyde dehydrogenase, mitochondrial
30	alpha-1a adrenergic receptor	ADRA1A			P35348	Alpha-1A adrenergic receptor
31	alpha-1b adrenergic receptor	ADRA1B			P35368	Alpha-1B adrenergic receptor
32	alpha-1d adrenergic receptor	ADRA1D	ADRA1A		P25100	Alpha-1D adrenergic receptor
33	alpha-2a adrenergic receptor [homo sapiens]	ADRA2A			P08913	Alpha-2A adrenergic receptor
34	angiotensin ii receptor	AGTR2			P50052	Type-2 angiotensin II receptor
35	angiotensin ii type 1a (at-1a)/1b (at-1b) receptor	AGTR1			P30556	Type-1 angiotensin II receptor
36	angiotensin-converting enzyme	ACE			P12821	Angiotensin-converting enzyme
37	cannabinoid cb1 receptor	CNR1			P21554	Cannabinoid receptor 1
38	cannabinoid cb1 receptor/orexin receptor 1 complex	CNR1	HCRTR1		P21554 / O43613	Cannabinoid receptor 1 / Orexin receptor type 1
39	cannabinoid receptor 1	CNR1			P21554	Cannabinoid receptor 1
40	carbonic anhydrase 2	CA2			P00918	Carbonic anhydrase 2
41	carbonic anhydrase 4	CA4			P22748	Carbonic anhydrase 4
42	corticotropin releasing factor receptor 1	CRHR1			P34998	Corticotropin-releasing factor receptor 1
43	corticotropin releasing factor-binding protein	CRHBP			P24387	Corticotropin-releasing factor-binding protein
44	gaba a alpha4beta2gamma2	GABRA4	GABRB2	GABRG2	P48169 / P47870 / P18507	Gamma-aminobutyric acid receptor subunits alpha-4 / beta-2 / gamma-2
45	gaba a receptor alpha-1/beta-1/gamma-2	GABRA1	GABRB1	GABRG2	P14867 / P18505 / P18507	Gamma-aminobutyric acid receptor subunits alpha-1 / beta-1 / gamma-2
46	gaba a receptor alpha-3/beta-2/gamma-2	GABRA3	GABRB2	GABRG2	P34903 / P47870 / P18507	Gamma-aminobutyric acid receptor subunits alpha-3 / beta-2 / gamma-2
47	gaba a receptor alpha-4/beta-3/gamma-2	GABRA4	GABRB3	GABRG2	P48169 / P28472 / P18507	Gamma-aminobutyric acid receptor subunits alpha-4 / beta-3 / gamma-2
48	gaba a receptor alpha-6/beta-2/gamma-2	GABRA6	GABRB2	GABRG2	Q16445 / P47870 / P18507	Gamma-aminobutyric acid receptor subunits alpha-6 / beta-2 / gamma-2
49	gaba receptor alpha-1/beta-1/gamma-2 subunit	GABRA1	GABRB1	GABRG2	P14867 / P18505 / P18507	Gamma-aminobutyric acid receptor subunits alpha-1 / beta-1 / gamma-2
50	gaba receptor alpha-1/beta-2/gamma-2 subunit	GABRA1	GABRB2	GABRG2	P14867 / P47870 / P18507	Gamma-aminobutyric acid receptor subunits alpha-1 / beta-2 / gamma-2
51	gaba receptor alpha-1/beta-3/gamma-2 subunit	GABRA1	GABRB3	GABRG2	P14867 / P28472 / P18507	Gamma-aminobutyric acid receptor subunits alpha-1 / beta-3 / gamma-2
52	gaba receptor alpha-2/beta-2/gamma-2 subunit	GABRA2	GABRB2	GABRG2	P47869 / P47870 / P18507	Gamma-aminobutyric acid receptor subunits alpha-2 / beta-2 / gamma-2
53	gaba receptor alpha-2/beta-3/gamma-2 subunit	GABRA2	GABRB3	GABRG2	P47869 / P28472 / P18507	Gamma-aminobutyric acid receptor subunits alpha-2 / beta-3 / gamma-2
54	gaba receptor alpha-3/beta-2/gamma-2 subunit	GABRA3	GABRB2	GABRG2	P34903 / P47870 / P18507	Gamma-aminobutyric acid receptor subunits alpha-3 / beta-2 / gamma-2
55	gaba receptor alpha-3/beta-3/gamma-2 subunit	GABRA3	GABRB3	GABRG2	P34903 / P28472 / P18507	Gamma-aminobutyric acid receptor subunits alpha-3 / beta-3 / gamma-2
56	gaba receptor alpha-4/beta-3/gamma-2 subunit	GABRA4	GABRB3	GABRG2	P48169 / P28472 / P18507	Gamma-aminobutyric acid receptor subunits alpha-4 / beta-3 / gamma-2
57	gaba receptor alpha-5/beta-3/gamma-2 subunit	GABRA5	GABRB3	GABRG2	P31644 / P28472 / P18507	Gamma-aminobutyric acid receptor subunits alpha-5 / beta-3 / gamma-2
58	gaba receptor alpha-6/beta-3/gamma-2 subunit	GABRA5	GABRB3	GABRG2	Q16445 / P28472 / P18507	Gamma-aminobutyric acid receptor subunits alpha-6 / beta-3 / gamma-2
59	gaba receptor beta-3 subunit	GABRB3			P28472	Gamma-aminobutyric acid receptor subunit beta-3
60	gaba receptor rho-2 subunit	GABRR2			P28476	Gamma-aminobutyric acid receptor subunit rho-2
61	glutamate [nmda] receptor subunit epsilon 1/zeta 1	GRIN2A	GRIN1		Q12879 / Q05586	Glutamate receptor ionotropic, NMDA 2A / NMDA 1
62	glutamate [nmda] receptor subunit epsilon 2	GRIN2B			Q13224	Glutamate receptor ionotropic, NMDA 2B
63	glutamate [nmda] receptor subunit epsilon 2/zeta 1	GRIN2B	GRIN1		Q13224 / Q05586	Glutamate receptor ionotropic, NMDA 2B / NMDA 1
64	glutamate [nmda] receptor subunit epsilon 4	GRIN2D			O15399	Glutamate receptor ionotropic, NMDA 2D
65	glutamate [nmda] receptor	—			—	Glutamate receptor ionotropic, NMDA
66	glyceraldehyde-3-phosphate dehydrogenase liver	GAPDH			P04406	Glyceraldehyde-3-phosphate dehydrogenase
67	glycine receptor (alpha-1/beta)	GLRA1	GLRB		P23415 / P48167	Glycine receptor subunits alpha-1 / beta
68	histamine h1 receptor	HRH1			P35367	Histamine H1 receptor
69	histamine h2 receptor	HRH2			P25021	Histamine H2 receptor
70	insulin receptor	INSR			P06213	Insulin receptor
71	melatonin receptor	MTNR1A			P48039	Melatonin receptor type 1A
72	mitochondrial complex i (nadh dehydrogenase)	—			—	**NADH dehydrogenase**
73	muscarinic acetylcholine receptor	CHRM1			P11229	Muscarinic acetylcholine receptor M1
74	nadh-ubiquinone oxidoreductase mlrq subunit	NDUFA4			O00483	Cytochrome c oxidase subunit NDUFA4
75	opioid receptors; mu/kappa/delta	OPRM1	OPRK1	OPRD1	P35372 / P41145 / P41143	Mu-type / Kappa-type / Delta-type opioid receptor
76	potassium channel subfamily k member 9	KCNK9			Q9NPC2	Potassium channel subfamily K member 9
77	progesterone	PGR			P06401	Progesterone receptor
78	secretin receptor	SCTR			P47872	Secretin receptor
79	sodium channel protein type i alpha subunit	SCN1A			P35498	Sodium channel protein type 1 subunit alpha
80	sodium channel protein type i i alpha subunit	SCN2A			Q99250	Sodium channel protein type 2 subunit alpha
81	sodium channel protein type iii alpha subunit	SCN3A			Q9NY46	Sodium channel protein type 3 subunit alpha
82	sodium channel protein type xi alpha subunit	SCN9A			Q15858	Sodium channel protein type 9 subunit alpha
83	synaptic vesicular amine transporter	SLC18A2			Q05940	Synaptic vesicular amine transporter
84	tacr1	TACR1			P25103	Substance-P receptor
85	vasopressin v1 receptor	AVPR1B			P47901	Vasopressin V1b receptor
86	vasopressin v1a receptor (v1a)	AVPR1A			P37288	Vasopressin V1a receptor
87	voltage-gated calcium channel	—			—	Voltage-dependent L-type calcium channel
88	voltage-gated l-type calcium channel alpha-1c subunit	CACNA1C			Q13936	Voltage-dependent L-type calcium channel subunit alpha-1C
89	voltage-gated l-type calcium channel					
90	voltage-gated n-type calcium channel alpha-1b subunit	CACNA1B			Q00975	Voltage-dependent N-type calcium channel subunit alpha-1B
91	voltage-gated t-type calcium channel alpha-1g subunit	CACNA1G			O43497	Voltage-dependent T-type calcium channel subunit alpha-1G
92	voltage-gated t-type calcium channel	—			—	Voltage-dependent L-type calcium channel
