# Supplementary material for: Consensus Ensemble Multitarget Neural Network Model of Anxiolytic Activity of Chemical Compounds and Its Use for Multitarget Pharmacophore Design
Source: Pharmaceuticals (Basel). 2023 May 11;16(5):731. doi: 10.3390/ph16050731 (PMC10223244; doi:10.3390/ph16050731)
Supplement: Supplementary file 1 [file pharmaceuticals-16-00731-s001.zip › TableS2 Anxiolytic Chemotypes.pdf]

Table S2. The chemopypes of the studied derivatives of nitrogen-containing condensed heterocycles.

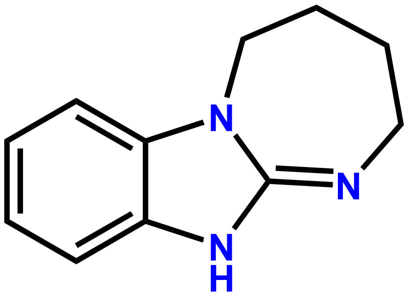<br>2,3,4,5-tetrahydro-1H-[1,3]diazepino[1,2-a]benzimidazole<br>Chemotype weight: 70	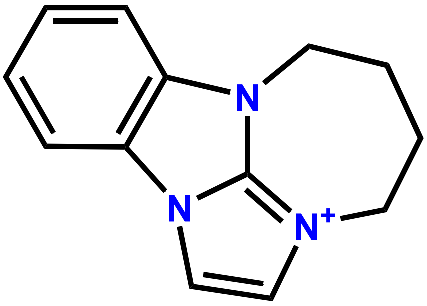<br>1,4-diaza-9-azoniatetracyclo[7.6.1.04,16.010,15]hexadeca-2,9(16),10,12,14-pentaene<br>Chemotype weight: 6	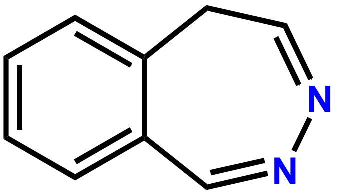<br>5H-2,3-benzodiazepine<br>Chemotype weight: 11
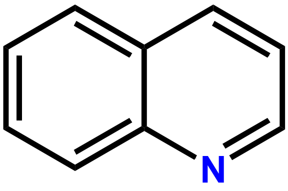<br>Quinoline<br>Chemotype weight: 10	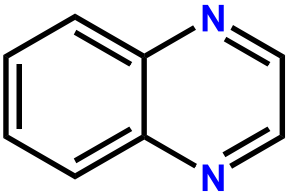<br>Quinoxaline<br>Chemotype weight: 25	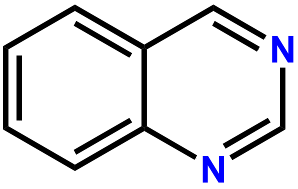<br>Quinazoline<br>Chemotype weight: 10
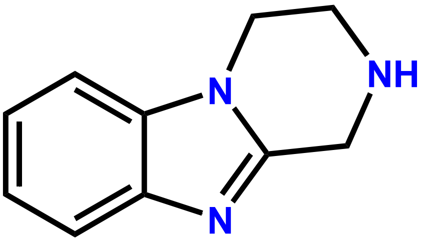<br>1,2,3,4-tetrahydropyrazino[1,2-a]benzimidazole<br>Chemotype weight: 15	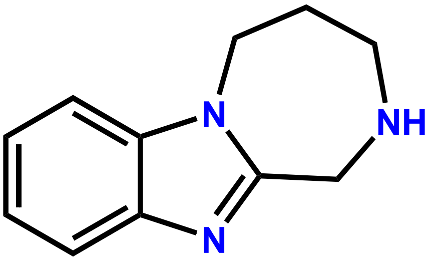<br>2,3,4,5-tetrahydro-1H-[1,4]diazepino[1,2-a]benzimidazole<br>Chemotype weight: 5	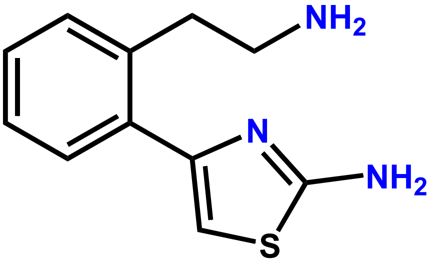<br>4-[2-(2-aminoethyl)phenyl]-1,3-thiazol-2-amine<br>Chemotype weight: 10
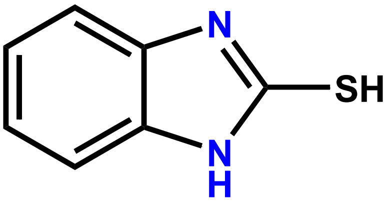<br>1,3-dihydrobenzimidazole-2-thione<br>Chemotype weight: 12	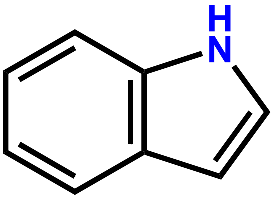<br>1H-indole<br>Chemotype weight: 5	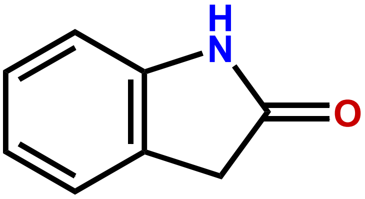<br>Oxindole<br>Chemotype weight: 5
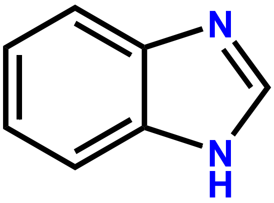<br>1H-benzimidazole<br>Chemotype weight: 10	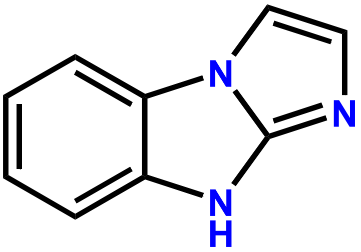<br>9H-imidazo[1,2-a]benzimidazole<br>Chemotype weight: 10	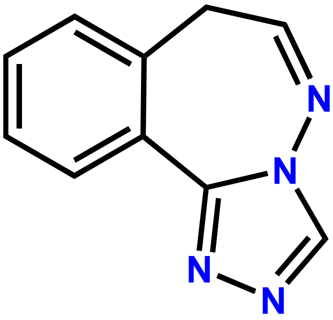<br>7H-[1,2,4]triazolo[3,4-a][2,3]benzodiazepine<br>Chemotype weight: 10
