# Supplementary material for: Consensus Ensemble Multitarget Neural Network Model of Anxiolytic Activity of Chemical Compounds and Its Use for Multitarget Pharmacophore Design
Source: Pharmaceuticals (Basel). 2023 May 11;16(5):731. doi: 10.3390/ph16050731 (PMC10223244; doi:10.3390/ph16050731)
Supplement: Supplementary file 1 [file pharmaceuticals-16-00731-s001.zip › TableS3 14Rel_Targets.pdf]

Table S3. Significant anxiolytic biotargets of chemotypes of nitrogen-containing heterocycles.

Target	Genes	Ind_{Gen}[1]	Ind_{Max}[2]	Chemotype
Synaptic vesicular amine transporter (VMAT2)	SLC18A2	2.50	2.50	Diazepinobenzimidazole
5-hydroxytryptamine receptor 1A	HTR1A	1.95	2.80	Benzodiazepine
Melatonin receptor type 1A (MT1)	MTNR1A	1.15	2.69	Oxindole
Melatonin receptor type 1B (MT2)	MTNR1B	1.15	2.64	Imidazobenzimidazole
NMDA receptor GluN1/GluN2A/GluN2B	GRIN1/GRIN2A/GRIN2B	1.05	3.80	Diazepinobenzimidazole
Alpha-2B adrenergic receptor	ADRA2B	1.04	2.65	Diazepinobenzimidazole
5-hydroxytryptamine receptor 7	HTR7	1.00	1.00	Diazepinobenzimidazole
Type-1 angiotensin II receptor	AGTR1	1.00	1.00	Diazepinobenzimidazole
Alpha-1A adrenergic receptor	ADRA1A	1.00	1.00	Diazepinobenzimidazole
Alpha-1B adrenergic receptor	ADRA1B	1.00	1.00	Diazepinobenzimidazole
Alpha-2A adrenergic receptor	ADRA2A	1.00	1.00	Diazepinobenzimidazole
GABA-A receptor alpha1-beta2-gamma2	GABRA1/GABRB2/GABRG2	0.65	3.82	Benzimidazole
5-hydroxytryptamine receptor 4	HTR4	0.03	3.27	Triazabenzocyclopentaazulenium
5-hydroxytryptamine receptor 2A	HTR2A	-0.68	2.60	Quinoline

[1] The general average value of the activity level index for all chemotypes.

[2] The maximum average value of the activity level index for a given chemotype.
