# Supplementary material for: Consensus Ensemble Multitarget Neural Network Model of Anxiolytic Activity of Chemical Compounds and Its Use for Multitarget Pharmacophore Design
Source: Pharmaceuticals (Basel). 2023 May 11;16(5):731. doi: 10.3390/ph16050731 (PMC10223244; doi:10.3390/ph16050731)
Supplement: Supplementary file 1 [file pharmaceuticals-16-00731-s001.zip › TableS4 Num_3Dmodels.pdf]

Table S4. Numbers of 3D models of anxiolytic biotargets relevant to chemotypes of nitrogen-containing heterocycles.

Target	Name	X-Ray[1]	Model[2]
SLC18A2	Synaptic vesicular amine transporter (VMAT2)	0	1
HTR1A	5-hydroxytryptamine receptor 1A	3	1
MTNR1A	Melatonin receptor type 1A (MT1)	4	1
MTNR1B	Melatonin receptor type 1B (MT2)	4	1
GRIN1/GRIN2A/GRIN2B	NMDA receptor GluN1/GluN2A/GluN2B	1	0
ADRA2B	Alpha-2B adrenergic receptor	2	1
HTR7	5-hydroxytryptamine receptor 7	0	1
AGTR1	Type-1 angiotensin II receptor	6	1
ADRA1A	Alpha-1A adrenergic receptor	0	1
ADRA1B	Alpha-1B adrenergic receptor	0	1
ADRA2A	Alpha-2A adrenergic receptor	2	1
GABRA1/GABRB2/GABRG2	GABA-A receptor alpha1-beta2-gamma2	2	1
HTR4	5-hydroxytryptamine receptor 4	0	1
HTR2A	5-hydroxytryptamine receptor 2A	5	2
Total		29	14

[1] Number of experimental models found in PDBe and RCSB PDB.
[2] Number of theoretical models constructed by homology modeling.
