# Supplementary material for: Consensus Ensemble Multitarget Neural Network Model of Anxiolytic Activity of Chemical Compounds and Its Use for Multitarget Pharmacophore Design
Source: Pharmaceuticals (Basel). 2023 May 11;16(5):731. doi: 10.3390/ph16050731 (PMC10223244; doi:10.3390/ph16050731)
Supplement: Supplementary file 1 [file pharmaceuticals-16-00731-s001.zip › TableS5 Val_3D models.pdf]

Table S5. Valid 3D models of biotargets relevant to the anxiolytic activity of chemotypes of nitrogen-containing heterocycles.

Target	Name	3D Model Code
GABRA1/GABRB2/GABRG2	GABA-A receptor alpha1-beta2-gamma2	6D6T
GRIN1/GRIN2A/GRIN2B	NMDA receptor GluN1/GluN2A/GluN2B	5UOW
HTR1A	5-hydroxytryptamine receptor 1A	7E2Y
HTR7	5-hydroxytryptamine receptor 7	AF-P34969-F1-model_v1
HTR4	5-hydroxytryptamine receptor 4	AF-Q13639-F1-model_v1
HTR2A	5-hydroxytryptamine receptor 2A	6A93
SLC18A2	Synaptic vesicular amine transporter (VMAT2)	AF-Q05940-F1-model_v1
AGTR1	Type-1 angiotensin II receptor	4YAY
ADRA1A	Alpha-1A adrenergic receptor	AF-P35348-F1-model_v1
ADRA1B	Alpha-1B adrenergic receptor	AF-P35368-F1-model_v1
ADRA2A	Alpha-2A adrenergic receptor	6KUX
ADRA2B	Alpha-2B adrenergic receptor	6K41
MTNR1A	Melatonin receptor type 1A (MT1)	6ME3
MTNR1B	Melatonin receptor type 1B (MT2)	6ME6
