# Supplementary material for: Consensus Ensemble Multitarget Neural Network Model of Anxiolytic Activity of Chemical Compounds and Its Use for Multitarget Pharmacophore Design
Source: Pharmaceuticals (Basel). 2023 May 11;16(5):731. doi: 10.3390/ph16050731 (PMC10223244; doi:10.3390/ph16050731)
Supplement: Supplementary file 1 [file pharmaceuticals-16-00731-s001.zip › TableS6 Sites_Targets.pdf]

Table S6. The key amino acids of binding sites of valid 3D models of biotargets relevant to the anxiolytic activity of chemotypes of nitrogen-containing heterocycles.

Target	3D Model Code	Key amino acides
GABRA1/GABRB2/GABRG2 GABA-binding	6D6T	Tyr97A, Glu155A, Tyr205A, Phe64B, Arg66B, Thr129B, Arg131B
GABRA1/GABRB2/GABRG2 benzodiazepine-binding	6D6T	Phe99D, His101D, Tyr159D, Phe77E, Met130E, Thr142E
GRIN1/GRIN2A/GRIN2B glutamate-binding	5UOW	His473, Thr501, Gly676, Ser677, Thr678, Asp719, Glu1101
GRIN1/GRIN2A/GRIN2B Ca-channel blocking	5UOW	Leu630, Ala631, Thr634, Leu636, Ala637, Thr640, Thr646, Leu649
HTR1A	7E2Y	Asp116, Cys120, Ser199, Phe361, Phe362, Tyr390
HTR7	AF-P34969-F1-model_v1	Val134, Phe137, Val138, Thr141, Phe158, Ile159, Ala160, Asp162, Val163, Trp340, Phe343, Phe344, Ser347, Arg350, Trp364, Glu366, Arg367, Leu370, Tyr374
HTR4	AF-Q13639-F1-model_v1	Trp86, Arg96, Asp100, Thr104, Tyr192, Ala193, Cys196, Ser197, Trp294
HTR2A serotonin-binding	6A93	Ala207, Tyr266, Val210, Val214, Leu211, Leu46, Thr42
HTR2A allosteric	6A93	Phe225, Val252, Trp222, Ile96, Phe218, Phe226
SLC18A2	AF-Q05940-F1-model_v1	Asp33, Asp262, Glu312, Tyr341, Asp399, Asp426, Asp460
AGTR1	4YAY	Tyr35, Trp84, Thr88, Ser105, Val108, Ser109, Leu112, Ala163, Arg167, Phe182, Pro285, Ile288, Tyr292
ADRA1A	AF-P35348-F1-model_v1	Asn44, Asp72, Ser83, Phe86, Trp102, Asp106, Val107, Cys110, Thr111, Ile157, Ser158, Pro161, Gln167, Ile178, Tyr184, Ser188, Ala189, Ser192, Phe193, Leu290, Phe288, Phe289, Phe308, Lys309, Phe312, Tyr316, Asn322, Tyr326, Ser330
ADRA1B	AF-P35368-F1-model_v1	Asn63, Asp91, Asp125, Thr130, Arg143, Ser207, Arg288, Lys291, Thr295, Phe310, Asn344, Thr348, Ser352
ADRA2A	6KUX	Tyr109, Asp113, Val114, Cys188, Glu189, Ile190, Phe390, Phe391, Phe408, Phe412, Tyr416
ADRA2B	6K41	Asp92, Val93, Cys96, Thr97, Leu166, Ser176, Ser180, Trp384, Phe387, Phe388, Tyr391, Phe412, Tyr 416
MTNR1A	6ME3	Ala104, Met107, Gly108, Val111, Val159, Asn162, Leu168, Thr178, Phe179, Gln181, Val191, Leu254, Asn255, Tyr281, Ala284, Tyr285
MTNR1B	6ME6	Ala117, Met120, Gly121, Val124, Asn175, Phe192, Gln194, Tyr200, Val204, Val205, Leu267, Asn268, Tyr294, Ala297, Tyr298
