# Supplementary material for: Consensus Ensemble Multitarget Neural Network Model of Anxiolytic Activity of Chemical Compounds and Its Use for Multitarget Pharmacophore Design
Source: Pharmaceuticals (Basel). 2023 May 11;16(5):731. doi: 10.3390/ph16050731 (PMC10223244; doi:10.3390/ph16050731)
Supplement: Supplementary file 1 [file pharmaceuticals-16-00731-s001.zip › TableS7 Training_Set_Anxio.pdf]

Table S7. Training set for building a consensus ensemble multitarget neural network model of anxiolytic activity of chemical compounds relevant to chemotypes of nitrogen-containing condensed heterocycles.

Mol_ID	Code	Levels	LevH	LevHM	LevA	ADRA1A	ADRA1B	ADRA2A	ADRA2B	AGTR1	GABA-A-GABA	GABA-A-Benz	HTR1A	HTR2A-Spec	HTR2A-Allo	HTR4	HTR7	MTNR1A	MTNR1B	NMDA-Glut	NMDA-Ca	SLC18A2	Sample1	Sample2	Sample3	Sample4	Sample5	Sample6	Sample7
1	CHEMBL323519	high	h	hm	a	-6.4	-7.7	-7.6	-5.6	-8.8	-6.6	-8.3	-7.8	-7.5	-8.9	-8.1	-7.1	-7.6	-6.9	-7.2	-7.0	-4.3	train	val	test	train	train	train	train
2	CHEMBL204240	high	h	hm	a	-7.5	-7.1	-7.6	-5.1	-7.8	-6.4	-8.5	-7.8	-6.8	-8.2	-8.2	-7.6	-8.6	-7.8	-6.7	-6.4	-4.1	train	train	val	test	train	train	train
3	CHEMBL314608	high	h	hm	a	-6.4	-7.3	-7.8	-5.6	-7.9	-6.3	-8.3	-7.9	-7.0	-8.4	-8.1	-7.7	-8.0	-8.5	-7.0	-6.2	-5.0	train	train	train	val	test	train	train
4	CHEMBL319178	high	h	hm	a	-8.1	-8.7	-10.4	-6.5	-9.7	-7.5	-10.1	-10.4	-8.5	-11.1	-9.7	-9.7	-9.6	-8.5	-8.6	-8.7	-5.8	train	train	train	train	val	test	train
5	CHEMBL153823	high	h	hm	a	-7.7	-7.0	-8.2	-9.2	-8.1	-6.5	-8.8	-7.7	-6.9	-8.6	-7.8	-8.6	-8.4	-8.9	-7.3	-7.4	-5.9	train	train	train	train	train	val	test
6	CHEMBL3246317	high	h	hm	a	-8.3	-8.2	-8.5	-8.1	-8.9	-7.7	-10.1	-9.1	-7.9	-9.7	-9.2	-9.4	-8.2	-9.6	-7.0	-7.2	-5.4	test	train	train	train	train	train	val
7	CHEMBL98258	high	h	hm	a	-8.5	-8.6	-10.6	-6.6	-9.6	-7.7	-10.4	-10.7	-8.7	-11.4	-10.2	-9.8	-8.7	-7.4	-8.4	-8.9	-5.9	val	test	train	train	train	train	train
8	CHEMBL155270	high	h	hm	a	-7.9	-7.2	-8.4	-8.9	-7.7	-6.2	-8.7	-7.7	-6.8	-8.4	-7.6	-8.4	-8.2	-8.5	-7.1	-7.4	-4.6	train	val	test	train	train	train	train
9	CHEMBL86611	high	h	hm	a	-7.3	-7.1	-7.2	-6.5	-7.3	-6.2	-7.7	-7.7	-6.5	-8.2	-7.8	-7.3	-7.9	-8.0	-6.7	-6.1	-4.6	train	train	val	test	train	train	train
10	CHEMBL87790	high	h	hm	a	-7.1	-6.9	-6.8	-6.7	-7.2	-6.1	-7.6	-7.1	-6.1	-8.0	-7.4	-7.2	-8.3	-8.0	-6.3	-5.7	-4.4	train	train	train	val	test	train	train
11	CHEMBL319966	high	h	hm	a	-9.0	-8.5	-9.4	-7.1	-9.0	-7.9	-9.3	-9.2	-7.6	-10.5	-8.7	-9.3	-9.9	-9.4	-7.5	-7.8	-5.1	train	train	train	train	val	test	train
12	CHEMBL88106	high	h	hm	a	-7.3	-7.1	-7.0	-6.0	-7.5	-6.4	-8.1	-7.7	-6.5	-8.2	-7.8	-7.5	-8.1	-8.1	-6.4	-6.1	-4.6	train	train	train	train	train	val	test
13	CHEMBL86557	high	h	hm	a	-7.6	-7.1	-7.5	-5.9	-7.8	-6.7	-8.0	-8.1	-6.8	-8.4	-8.0	-7.7	-8.7	-8.3	-6.6	-6.6	-4.7	test	train	train	train	train	train	val
14	CHEMBL2263293	high	h	hm	a	-8.3	-7.9	-8.6	-6.3	-8.6	-8.0	-9.6	-9.1	-8.0	-9.9	-8.6	-7.6	-7.5	-8.3	-7.4	-7.1	-4.5	val	test	train	train	train	train	train
15	CHEMBL20042	high	h	hm	a	-8.7	-8.4	-9.4	-6.1	-9.4	-7.0	-9.6	-9.1	-7.4	-10.0	-8.6	-7.9	-8.1	-9.3	-8.0	-7.6	-5.4	train	val	test	train	train	train	train
16	CHEMBL101304	high	h	hm	a	-7.4	-8.6	-7.7	-5.4	-8.3	-6.5	-8.4	-8.0	-7.5	-9.8	-8.3	-7.7	-9.5	-9.8	-6.9	-6.9	-4.4	train	train	val	test	train	train	train
17	CHEMBL2263296	high	h	hm	a	-8.3	-8.0	-9.5	-6.7	-9.5	-8.7	-9.9	-9.8	-8.3	-9.9	-8.5	-8.3	-8.2	-8.4	-7.7	-8.1	-5.3	train	train	train	val	test	train	train
18	CHEMBL260994	high	h	hm	a	-8.2	-8.6	-9.1	-6.3	-9.7	-7.5	-9.9	-9.5	-7.9	-9.8	-8.7	-8.8	-8.2	-8.8	-8.2	-8.2	-5.4	train	train	train	train	val	test	train
19	CHEMBL347801	high	h	hm	a	-8.0	-7.3	-8.6	-8.7	-8.2	-6.6	-9.0	-8.0	-7.1	-8.8	-8.0	-9.0	-8.7	-9.1	-7.5	-7.7	-4.8	train	train	train	train	train	val	test
20	CHEMBL4093093	high	h	hm	a	-8.3	-8.2	-8.9	-6.0	-9.6	-7.1	-9.5	-9.3	-8.2	-9.7	-8.6	-8.3	-8.7	-9.0	-7.7	-7.3	-5.2	test	train	train	train	train	train	val
21	CHEMBL661	high	h	hm	a	-8.7	-8.4	-8.6	-8.1	-9.3	-7.3	-10.6	-9.3	-8.2	-9.3	-9.5	-10.1	-9.2	-9.9	-7.1	-7.6	-5.5	val	test	train	train	train	train	train
22	CHEMBL562219	high	h	hm	a	-8.0	-7.4	-8.1	-5.1	-8.8	-6.4	-8.0	-8.1	-7.1	-8.9	-8.4	-8.5	-9.1	-9.5	-6.6	-6.7	-4.6	train	val	test	train	train	train	train
23	CHEMBL2263292	high	h	hm	a	-8.3	-7.9	-8.7	-6.4	-8.9	-7.3	-9.7	-9.1	-8.0	-9.8	-8.5	-7.9	-7.6	-8.2	-7.6	-7.5	-4.5	train	train	val	test	train	train	train
24	CHEMBL470786	high	h	hm	a	-7.5	-7.3	-8.2	-6.1	-8.3	-6.5	-8.8	-7.7	-7.1	-8.2	-8.1	-7.5	-7.7	-8.0	-7.3	-6.7	-4.4	train	train	train	val	test	train	train
25	CHEMBL469737	high	h	hm	a	-8.3	-7.3	-9.0	-6.1	-9.1	-7.1	-9.7	-8.1	-7.4	-11.1	-8.7	-6.8	-9.5	-7.0	-7.8	-7.6	-2.9	train	train	train	train	val	test	train
26	CHEMBL378298	high	h	hm	a	-8.0	-8.8	-9.4	-6.2	-9.0	-7.4	-9.4	-9.3	-8.6	-10.6	-8.9	-8.7	-8.8	-7.6	-7.8	-8.7	-2.9	train	train	train	train	train	val	test
27	CHEMBL646	high	h	hm	a	-8.9	-8.7	-8.6	-8.2	-9.1	-7.5	-10.2	-8.5	-8.6	-9.5	-9.2	-9.5	-9.0	-9.8	-7.2	-7.7	-5.8	test	train	train	train	train	train	val
28	Diazepam	high	h	hm	a	-7.0	-8.2	-8.4	-8.2	-8.8	-6.8	-9.4	-8.3	-7.5	-8.8	-8.5	-9.3	-9.2	-9.0	-7.4	-7.1	-4.9	val	test	train	train	train	train	train
29	Phenazepam	high	h	hm	a	-7.8	-8.0	-9.1	-8.3	-8.8	-6.9	-10.4	-8.1	-7.7	-8.7	-8.3	-9.3	-8.9	-9.7	-6.7	-7.1	-5.1	train	val	test	train	train	train	train
30	Tofisopam	high	h	hm	a	-6.4	-8.1	-7.5	-5.8	-8.4	-6.7	-8.4	-8.4	-7.4	-8.7	-9.2	-7.1	-8.1	-10.3	-6.9	-6.7	-3.3	train	train	val	test	train	train	train
31	CHEMBL470788	moderate	nh	hm	a	-8.1	-7.3	-7.5	-5.9	-8.4	-7.1	-8.9	-7.7	-7.2	-8.8	-8.8	-7.2	-6.7	-8.4	-7.1	-7.1	-4.6	train	train	train	val	test	train	train
32	CHEMBL471822	moderate	nh	hm	a	-7.8	-7.4	-8.5	-6.1	-8.3	-6.4	-8.6	-7.8	-6.9	-9.1	-8.6	-7.5	-7.9	-7.8	-7.4	-7.0	-4.3	train	train	train	train	val	test	train
33	CHEMBL470787	moderate	nh	hm	a	-8.3	-8.3	-8.3	-7.6	-8.5	-7.2	-9.3	-8.6	-7.4	-9.3	-8.7	-7.8	-7.9	-9.1	-7.6	-7.6	-5.0	train	train	train	train	train	val	test
34	CHEMBL204040	moderate	nh	hm	a	-7.2	-7.1	-7.7	-5.3	-7.8	-6.3	-8.5	-7.6	-7.0	-8.4	-8.0	-7.6	-8.4	-7.9	-6.6	-6.3	-4.1	test	train	train	train	train	train	val
35	CHEMBL154043	moderate	nh	hm	a	-7.4	-7.4	-8.3	-8.7	-7.7	-6.7	-8.6	-7.6	-6.9	-8.4	-7.8	-8.3	-8.1	-8.5	-7.2	-7.0	-5.8	val	test	train	train	train	train	train
36	CHEMBL283993	moderate	nh	hm	a	-8.0	-7.8	-8.9	-6.0	-9.4	-7.0	-9.5	-8.3	-7.4	-10.9	-9.0	-8.4	-8.8	-8.5	-7.5	-7.7	-5.2	train	val	test	train	train	train	train
37	CHEMBL314823	moderate	nh	hm	a	-6.8	-6.7	-6.9	-7.2	-7.0	-5.8	-8.0	-7.3	-6.2	-7.5	-7.5	-7.0	-7.4	-7.5	-6.2	-5.6	-4.6	train	train	val	test	train	train	train
38	CHEMBL315419	moderate	nh	hm	a	-6.9	-7.0	-6.9	-5.7	-7.0	-5.9	-7.7	-7.4	-6.3	-7.7	-7.3	-6.8	-7.3	-7.4	-6.2	-5.6	-4.3	train	train	train	val	test	train	train
39	CHEMBL3246236	moderate	nh	hm	a	-7.0	-7.5	-7.7	-7.7	-8.2	-6.5	-9.1	-8.0	-7.5	-9.3	-8.3	-8.2	-9.0	-8.6	-7.3	-7.2	-4.5	train	train	train	train	val	test	train
40	CHEMBL328250	moderate	nh	hm	a	-8.1	-8.5	-8.3	-6.0	-9.1	-7.0	-9.9	-9.2	-7.6	-9.0	-8.0	-7.0	-8.0	-8.7	-6.9	-7.0	-5.5	train	train	train	train	train	val	test
41	CHEMBL411663	moderate	nh	hm	a	-8.6	-8.3	-9.2	-6.4	-9.6	-7.1	-10.0	-9.5	-8.2	-10.2	-8.6	-9.0	-8.4	-9.3	-8.1	-8.2	-5.2	test	train	train	train	train	train	val
42	CHEMBL420977	moderate	nh	hm	a	-7.2	-7.0	-6.9	-6.5	-7.1	-6.2	-7.8	-7.4	-6.3	-7.9	-7.6	-7.3	-7.9	-7.9	-6.3	-5.9	-4.6	val	test	train	train	train	train	train
43	CHEMBL431020	moderate	nh	hm	a	-7.1	-6.9	-7.3	-6.5	-7.4	-6.1	-7.9	-7.6	-6.5	-7.9	-7.7	-7.3	-7.7	-8.1	-6.4	-6.1	-4.6	train	val	test	train	train	train	train
44	CHEMBL523	moderate	nh	hm	a	-7.9	-8.3	-8.2	-8.0	-8.5	-6.8	-9.6	-8.3	-7.1	-8.7	-8.6	-9.4	-9.4	-9.1	-7.0	-7.3	-4.7	train	train	val	test	train	train	train
45	CHEMBL83085	moderate	nh	hm	a	-7.2	-6.9	-7.3	-6.3	-7.5	-6.1	-7.5	-7.7	-6.4	-8.1	-7.9	-7.0	-8.1	-8.0	-6.4	-5.9	-4.4	train	train	train	val	test	train	train
46	CHEMBL83349	moderate	nh	hm	a	-7.0	-7.1	-7.0	-6.4	-7.1	-6.1	-7.7	-7.4	-6.3	-7.9	-7.6	-6.9	-7.9	-7.6	-6.2	-5.8	-4.2	train	train	train	train	val	test	train
47	CHEMBL86463	moderate	nh	hm	a	-6.9	-7.0	-6.9	-6.4	-7.2	-5.8	-7.7	-7.2	-6.3	-7.8	-7.3	-6.7	-8.0	-7.8	-6.4	-5.6	-4.6	train	train	train	train	train	val	test
48	CHEMBL87700	moderate	nh	hm	a	-7.3	-7.0	-7.2	-6.4	-7.6	-6.4	-8.0	-7.8	-6.5	-8.2	-7.7	-7.5	-8.1	-8.0	-6.7	-6.1	-4.7	test	train	train	train	train	train	val
49	CHEMBL98848	moderate	nh	hm	a	-9.2	-8.7	-9.6	-7.4	-9.2	-7.6	-10.4	-9.4	-8.3	-10.8	-9.1	-9.0	-9.6	-8.8	-7.9	-8.3	-5.3	val	test	train	train	train	train	train
50	CHEMBL969	moderate	nh	hm	a	-8.6	-7.8	-8.2	-8.1	-8.8	-6.8	-10.0	-9.5	-7.5	-9.1	-8.1	-8.2	-9.7	-10.3	-6.6	-7.5	-5.3	train	val	test	train	train	train	train
51	CHEMBL431105	moderate	nh	hm	a	-7.5	-7.6	-8.7	-5.7	-8.4	-7.3	-9.1	-8.0	-8.0	-9.2	-8.0	-8.0	-8.0	-7.5	-6.8	-7.0	-4.1	train	train	val	test	train	train	train
52	CHEMBL204755	moderate	nh	hm	a	-7.7	-7.1	-7.9	-5.3	-7.5	-6.5	-8.5	-7.5	-6.6	-8.4	-7.7	-7.5	-8.5	-7.9	-6.8	-6.5	-4.2	train	train	train	val	test	train	train
53	CHEMBL383127	moderate	nh	hm	a	-7.9	-7.3	-8.5	-6.7	-7.5	-6.7	-9.4	-8.0	-6.8	-8.4	-7.9	-8.2	-8.8	-8.3	-7.2	-7.0	-4.2	train	train	train	train	val	test	train
54	CHEMBL87271	moderate	nh	hm	a	-6.7	-6.8	-6.9	-6.4	-7.1	-5.9	-7.7	-7.0	-6.3	-7.7	-7.2	-6.8	-8.0	-7.9	-6.3	-5.9	-5.1	train	train	train	train	train	val	test
55	CHEMBL410628	moderate	nh	hm	a	-8.3	-7.9	-8.4	-6.1	-9.3	-7.1	-9.7	-9.2	-7.8	-9.9	-8.3	-8.0	-8.1	-8.8	-8.1	-8.5	-4.8	test	train	train	train	train	train	val
56	CHEMBL83570	moderate	nh	hm	a	-6.5	-6.7	-7.0	-6.0	-7.4	-6.1	-7.8	-7.2	-6.7	-7.9	-7.5	-6.8	-7.6	-7.7	-6.5	-5.9	-4.1	val	test	train	train	train	train	train
57	CHEMBL87662	moderate	nh	hm	a	-6.9	-7.1	-7.1	-6.3	-7.3	-5.9	-7.7	-7.5	-6.1	-7.6	-7.4	-6.8	-7.7	-7.8	-6.2	-5.8	-4.6	train	val	test	train	train	train	train
58	CHEMBL346817	moderate	nh	hm	a	-8.0	-7.2	-8.7	-9.0	-8.2	-6.7	-9.3	-8.0	-6.8	-8.6	-8.1	-9.0	-8.6	-9.0	-7.5	-7.5	-5.9	train	train	val	test	train	train	train
59	CHEMBL153953	moderate	nh	hm	a	-8.0	-6.9	-8.1	-7.8	-7.5	-6.1	-8.3	-7.5	-6.7	-8.2	-7.5	-8.5	-8.2	-8.5	-7.0	-7.2	-4.6	train	train	train	val	test	train	train
60	CHEMBL347113	moderate	nh	hm	a	-8.1	-7.0	-8.4	-8.1	-7.9	-6.5	-8.7	-8.0	-6.7	-8.7	-8.0	-9.0	-8.6	-9.1	-7.4	-7.5	-4.9	train	train	train	train	val	test	train
61	CHEMBL84698	moderate	nh	hm	a	-6.3	-6.7	-6.9	-6.0	-7.4	-6.0	-7.8	-7.2	-6.4	-7.8	-7.6	-6.8	-7.5	-7.6	-6.1	-5.9	-4.3	train	train	train	train	train	val	test
62	CHEMBL315080	moderate	nh	hm	a	-6.9	-7.3	-7.1	-6.5	-7.3	-6.1	-7.9	-7.7	-6.5	-8.2	-7.4	-7.0	-8.4	-8.2	-6.5	-6.1	-4.7	test	train	train	train	train	train	val
63	CHEMBL471821	moderate	nh	hm	a	-7.8	-8.2	-8.4	-7.0	-8.6	-7.3	-8.9	-9.0	-7.3	-9.2	-8.7	-8.1	-8.1	-9.1	-7.6	-7.5	-4.7	val	test	train	train	train	train	train
64	CHEMBL276971	moderate	nh	hm	a	-7.9	-7.7	-9.3	-6.2	-8.9	-6.9	-8.4	-8.5	-7.6	-9.3	-8.7	-7.8	-8.2	-8.0	-7.7	-7.7	-4.8	train	val	test	train	train	train	train

65	CHEMBL314033	moderate	nh	hm	a	-6.7	-6.8	-7.1	-6.2	-7.2	-5.7	-7.7	-7.2	-6.6	-7.9	-7.6	-6.9	-7.8	-7.6	-6.2	-6.1	-4.3	train	train	val	test	train	train	train
66	CHEMBL99303	moderate	nh	hm	a	-8.6	-8.6	-10.0	-6.7	-9.3	-7.7	-9.6	-10.8	-8.7	-11.4	-9.7	-8.9	-8.1	-7.1	-8.2	-8.6	5.9	train	train	train	val	test	train	train
67	CHEMBL345344	moderate	nh	hm	a	-7.8	-6.8	-8.0	-9.0	-7.5	-6.0	-8.5	-7.3	-6.6	-8.1	-7.3	-8.0	-7.9	-8.3	-7.0	-7.0	-5.6	train	train	train	train	val	test	train
68	CHEMBL87973	moderate	nh	hm	a	-7.2	-6.9	-7.3	-6.0	-7.4	-6.3	-7.8	-7.8	-6.5	-8.1	-7.7	-7.1	-8.5	-7.9	-6.5	-6.3	-4.5	train	train	train	train	train	val	test
69	CHEMBL207493	moderate	nh	hm	a	-7.3	-8.5	-8.7	-6.6	-9.3	-7.1	-9.0	-8.9	-8.5	-9.6	-9.0	-8.3	-8.5	-7.9	-7.8	-8.4	-4.2	test	train	train	train	train	train	val
70	CHEMBL231825	moderate	nh	hm	a	-7.7	-9.5	-9.7	-5.8	-9.9	-7.7	-9.4	-9.8	-9.6	-10.3	-9.4	-8.8	-8.7	-7.1	-8.4	-8.8	-2.9	val	test	train	train	train	train	train
71	CHEMBL87598	moderate	nh	hm	a	-6.7	-6.9	-7.0	-5.2	-7.7	-5.9	-8.1	-7.4	-6.8	-7.8	-7.5	-7.0	-7.9	-8.0	-6.0	-6.3	-4.6	train	val	test	train	train	train	train
72	CHEMBL261209	moderate	nh	hm	a	-8.1	-8.6	-9.0	-6.3	-9.7	-7.3	-10.1	-9.6	-8.2	-9.9	-8.9	-9.5	-8.6	-8.9	-8.4	-8.3	-5.4	train	train	val	test	train	train	train
73	CHEMBL2263295	moderate	nh	hm	a	-7.9	-7.7	-9.0	-6.1	-9.0	-8.4	-9.6	-8.7	-7.8	-9.3	-7.9	-8.5	-8.0	-8.2	-7.4	-7.3	-6.0	train	train	train	val	test	train	train
74	CHEMBL86761	moderate	nh	hm	a	-6.4	-6.7	-7.1	-5.1	-7.3	-5.8	-7.3	-7.3	-6.3	-7.7	-7.5	-7.3	-7.2	-7.1	-6.0	-6.0	-4.1	train	train	train	train	val	test	train
75	CHEMBL2263297	moderate	nh	hm	a	-7.7	-7.6	-8.7	-6.0	-8.8	-8.4	-9.6	-8.7	-7.6	-9.1	-7.7	-7.8	-7.1	-7.3	-7.3	-7.2	-5.5	train	train	train	train	train	val	test
76	CHEMBL2263294	moderate	nh	hm	a	-8.2	-7.7	-8.6	-6.6	-8.6	-8.5	-9.9	-9.1	-8.0	-9.8	-8.2	-8.1	-7.8	-7.8	-7.8	-7.8	-4.7	test	train	train	train	train	train	val
77	CHEMBL2263298	moderate	nh	hm	a	-8.3	-7.8	-8.6	-6.1	-8.7	-8.2	-9.4	-8.3	-7.9	-9.4	-7.9	-8.4	-8.5	-8.6	-7.6	-7.2	-5.6	val	test	train	train	train	train	train
78	CHEMBL445	moderate	nh	hm	a	-8.8	-8.1	-8.1	-8.5	-8.7	-7.4	-8.1	-8.2	-7.6	-8.9	-8.3	-9.3	-9.4	-10.1	-6.7	-7.8	-4.5	train	val	test	train	train	train	train
79	CHEMBL87115	moderate	nh	hm	a	-6.8	-6.6	-7.3	-8.0	-7.2	-5.8	-7.6	-7.1	-6.4	-7.4	-7.0	-7.5	-7.7	-7.4	-6.2	-5.7	-5.9	train	train	val	test	train	train	train
80	CHEMBL471820	moderate	nh	hm	a	-7.4	-7.5	-8.2	-6.2	-8.2	-6.9	-9.0	-7.9	-7.3	-8.8	-8.6	-7.2	-7.8	-7.6	-7.3	-6.9	-4.7	train	train	train	val	test	train	train
81	CHEMBL3277547	moderate	nh	hm	a	-7.5	-7.6	-7.8	-5.4	-7.4	-7.0	-9.0	-7.6	-7.2	-8.0	-7.7	-7.5	-8.6	-8.1	-6.5	-6.8	-4.4	train	train	train	train	val	test	train
82	CHEMBL4098854	moderate	nh	hm	a	-7.9	-9.9	-9.1	-6.8	-9.3	-8.2	-10.1	-8.8	-8.9	-10.2	-9.6	-8.8	-7.9	-9.8	-8.1	-8.1	-4.6	train	train	train	train	train	val	test
83	CHEMBL4099053	moderate	nh	hm	a	-7.6	-8.4	-8.5	-5.9	-8.2	-7.0	-8.0	-8.2	-8.1	-9.4	-9.1	-6.6	-8.4	-8.1	-7.8	-7.1	-5.6	test	train	train	train	train	train	val
84	CHEMBL585105	moderate	nh	hm	a	-7.9	-8.7	-9.2	-6.6	-8.8	-7.3	-9.7	-9.9	-8.4	-10.5	-8.9	-8.1	-8.9	-7.0	-7.9	-8.0	-4.9	val	test	train	train	train	train	train
85	CHEMBL509913	moderate	nh	hm	a	-9.7	-9.5	-9.8	-6.9	-9.7	-7.8	-10.8	-10.0	-8.5	-11.3	-10.7	-8.7	-8.6	-8.6	-8.9	-8.3	-5.7	train	val	test	train	train	train	train
86	CHEMBL513711	moderate	nh	hm	a	-7.1	-7.0	-9.1	-6.7	-9.0	-7.0	-9.2	-8.5	-7.7	-11.1	-8.6	-7.2	-9.0	-6.8	-7.9	-7.8	-1.2	train	train	val	test	train	train	train
87	CHEMBL86304	moderate	nh	hm	a	-7.1	-6.6	-7.3	-6.4	-7.2	-6.0	-8.0	-7.1	-6.4	-7.7	-7.2	-7.1	-7.4	-7.4	-6.3	-6.1	-4.0	train	train	train	val	test	train	train
88	CHEMBL204091	moderate	nh	hm	a	-7.8	-7.0	-7.5	-5.5	-7.9	-6.1	-8.8	-7.7	-6.6	-8.3	-7.5	-7.4	-8.8	-8.9	-6.8	-6.4	-4.1	train	train	train	train	val	test	train
89	CHEMBL517725	moderate	nh	hm	a	-7.9	-10.0	-9.8	-6.3	-9.7	-8.1	-10.2	-9.9	-8.4	-9.7	-9.9	-7.7	-11.4	-9.9	-8.5	-8.8	-5.2	train	train	train	train	train	val	test
90	CHEMBL1949963	moderate	nh	hm	a	-8.2	-8.6	-9.4	-6.4	-8.9	-7.4	-10.0	-9.3	-8.6	-10.8	-9.2	-9.1	-8.7	-9.7	-8.1	-8.1	-5.0	test	train	train	train	train	train	val
91	CHEMBL4068928	moderate	nh	hm	a	-8.1	-9.8	-9.3	-6.9	-9.6	-8.6	-10.4	-9.4	-9.3	-10.1	-9.4	-9.0	-10.5	-10.9	-8.2	-8.6	-4.7	val	test	train	train	train	train	train
92	CHEMBL443986	moderate	nh	hm	a	-6.9	-7.5	-8.5	-5.5	-8.0	-6.4	-9.1	-8.0	-7.1	-8.1	-8.0	-6.8	-7.1	-8.6	-7.2	-6.3	-4.2	train	val	test	train	train	train	train
93	CHEMBL49	moderate	nh	hm	a	-7.7	-8.8	-8.1	-6.2	-8.8	-7.0	-8.8	-8.8	-8.3	-9.5	-8.7	-7.9	-7.3	-7.9	-7.5	-7.8	-4.6	train	train	val	test	train	train	train
94	Afobazol	moderate	nh	hm	a	-6.5	-6.5	-7.2	-5.0	-7.5	-5.9	-7.3	-7.1	-6.7	-7.4	-7.1	-7.6	-7.0	-7.6	-6.2	-6.0	-4.1	train	train	train	val	test	train	train
95	CHEMBL315315	low	nh	nhm	a	-5.8	-6.3	-6.3	-6.7	-7.0	-5.4	-7.5	-6.9	-6.1	-7.3	-7.2	-6.5	-7.0	-7.2	-5.9	-5.5	-3.9	train	train	train	train	val	test	train
96	CHEMBL315768	low	nh	nhm	a	-6.2	-6.4	-6.8	-6.9	-7.1	-5.6	-7.4	-7.0	-6.2	-7.4	-7.1	-6.5	-7.6	-7.6	-6.2	-5.6	-4.4	train	train	train	train	train	val	test
97	CHEMBL412630	low	nh	nhm	a	-6.0	-7.0	-6.9	-4.8	-7.5	-5.8	-6.6	-7.0	-6.8	-7.2	-7.3	-6.0	-8.4	-7.8	-5.8	-5.6	-3.6	test	train	train	train	train	train	val
98	CHEMBL204581	low	nh	nhm	a	-8.5	-7.8	-8.9	-5.8	-8.5	-6.6	-9.3	-8.5	-7.1	-9.4	-8.1	-8.3	-9.6	-9.7	-7.7	-7.2	-4.9	val	test	train	train	train	train	train
99	CHEMBL203902	low	nh	nhm	a	-8.2	-7.5	-8.6	-5.7	-8.3	-6.2	-8.9	-7.9	-6.7	-9.2	-7.8	-8.0	-9.2	-9.0	-7.1	-7.3	-4.7	train	val	test	train	train	train	train
100	CHEMBL3144798	low	nh	nhm	a	-8.6	-7.4	-9.0	-7.6	-8.3	-6.6	-9.4	-8.8	-7.0	-9.1	-7.9	-8.6	-9.5	-9.2	-7.4	-7.6	-5.1	train	train	val	test	train	train	train
101	CHEMBL87509	low	nh	nhm	a	-7.3	-7.4	-7.2	-5.7	-7.7	-6.2	-8.0	-7.9	-6.9	-8.4	-7.9	-8.2	-8.0	-8.3	-6.6	-6.5	-4.4	train	train	train	val	test	train	train
102	CHEMBL314780	low	nh	nhm	a	-6.1	-6.1	-6.7	-6.8	-6.7	-5.6	-7.3	-6.8	-6.0	-7.3	-6.8	-7.4	-7.2	-7.6	-6.0	-5.2	-3.9	train	train	train	train	val	test	train
103	CHEMBL85228	low	nh	nhm	a	-6.5	-6.9	-7.0	-5.1	-7.4	-5.7	-7.6	-7.5	-6.6	-7.5	-7.5	-7.2	-7.3	-7.6	-6.0	-5.7	-4.2	train	train	train	train	train	val	test
104	CHEMBL413575	low	nh	nhm	a	-8.4	-7.7	-8.2	-7.2	-7.4	-6.6	-9.0	-7.9	-6.9	-8.8	-8.1	-7.7	-8.7	-8.1	-6.8	-7.0	-3.7	test	train	train	train	train	train	val
105	CHEMBL407263	low	nh	nhm	a	-9.0	-7.5	-9.9	-6.7	-9.6	-6.5	-10.8	-9.8	-7.5	-10.4	-8.3	-9.4	-8.9	-10.2	-8.1	-8.1	-5.5	val	test	train	train	train	train	train
106	CHEMBL87251	low	nh	nhm	a	-6.4	-6.6	-6.9	-6.3	-7.4	-6.2	-7.8	-7.6	-6.5	-8.1	-7.7	-7.0	-8.1	-8.0	-6.6	-6.1	-4.7	train	val	test	train	train	train	train
107	CHEMBL204081	low	nh	nhm	a	-7.7	-8.1	-7.8	-5.7	-8.0	-6.7	-8.7	-8.3	-7.3	-8.3	-7.7	-8.2	-8.3	-7.6	-6.7	-6.7	-4.3	train	train	val	test	train	train	train
108	CHEMBL204748	low	nh	nhm	a	-8.3	-7.8	-8.1	-6.0	-8.0	-6.8	-9.2	-8.5	-7.1	-9.4	-7.9	-8.2	-8.5	-8.6	-7.5	-7.0	-4.7	train	train	train	val	test	train	train
109	CHEMBL206675	low	nh	nhm	a	-7.5	-7.5	-7.5	-7.0	-7.8	-6.5	-8.6	-7.8	-7.3	-8.1	-7.8	-8.1	-8.3	-8.1	-6.9	-6.3	-4.9	train	train	train	train	val	test	train
110	CHEMBL204027	low	nh	nhm	a	-7.3	-6.9	-7.7	-5.1	-7.7	-6.1	-7.8	-7.6	-7.0	-7.8	-7.8	-7.5	-8.0	-8.1	-6.6	-5.9	-4.0	train	train	train	train	train	val	test
111	CHEMBL383066	low	nh	nhm	a	-7.6	-7.1	-8.0	-6.9	-7.8	-6.4	-8.9	-7.9	-6.6	-8.1	-7.7	-7.6	-8.3	-8.2	-6.9	-6.2	-4.2	test	train	train	train	train	train	val
112	CHEMBL203708	low	nh	nhm	a	-7.2	-7.2	-7.1	-7.0	-7.6	-6.1	-8.9	-7.7	-7.0	-8.2	-7.5	-7.3	-8.0	-8.2	-6.6	-6.7	-4.7	val	test	train	train	train	train	train
113	CHEMBL101817	low	nh	nhm	a	-8.1	-8.4	-9.3	-5.9	-9.3	-7.4	-10.0	-9.9	-8.1	-10.5	-9.5	-8.9	-9.3	-8.3	-8.2	-7.8	-5.3	train	val	test	train	train	train	train
114	CHEMBL82817	low	nh	nhm	a	-6.5	-6.7	-6.9	-5.7	-7.4	-5.8	-7.6	-7.5	-6.5	-7.8	-7.4	-6.5	-7.2	-7.4	-6.6	-5.7	-4.5	train	train	val	test	train	train	train
115	CHEMBL348305	low	nh	nhm	a	-7.7	-7.0	-8.2	-9.0	-7.5	-6.3	-8.5	-7.5	-6.9	-8.0	-7.6	-8.1	-7.9	-8.4	-7.1	-7.0	-5.4	train	train	train	val	test	train	train
116	CHEMBL3246237	low	nh	nhm	a	-7.8	-7.7	-7.9	-6.7	-8.1	-6.3	-9.2	-8.0	-7.3	-8.9	-7.8	-8.1	-8.3	-8.7	-7.1	-7.2	-4.3	train	train	train	train	val	test	train
117	CHEMBL383680	low	nh	nhm	a	-8.4	-7.6	-8.1	-6.9	-7.7	-6.6	-9.0	-7.9	-7.0	-8.5	-8.1	-8.4	-8.8	-8.3	-7.0	-7.1	-3.9	train	train	train	train	train	val	test
118	CHEMBL440575	low	nh	nhm	a	-7.5	-7.2	-7.8	-5.5	-7.5	-6.4	-8.8	-7.9	-6.7	-8.1	-7.9	-7.7	-8.1	-8.4	-7.0	-6.6	-4.2	test	train	train	train	train	train	val
119	CHEMBL442753	low	nh	nhm	a	-7.2	-7.3	-7.2	-6.2	-7.5	-6.1	-7.9	-7.8	-6.5	-8.2	-7.5	-7.7	-7.8	-7.4	-6.5	-6.1	-4.4	val	test	train	train	train	train	train
120	CHEMBL565423	low	nh	nhm	a	-7.3	-8.2	-8.8	-6.8	-8.9	-7.3	-9.0	-9.7	-8.4	-9.7	-9.3	-8.6	-7.8	-6.8	-7.6	-8.0	-4.2	train	val	test	train	train	train	train
121	CHEMBL316194	low	nh	nhm	a	-7.7	-8.2	-8.7	-5.7	-8.6	-6.8	-8.9	-8.5	-7.4	-8.9	-8.5	-8.0	-8.3	-8.3	-7.4	-7.2	-5.1	train	train	val	test	train	train	train
122	CHEMBL3246239	low	nh	nhm	a	-7.4	-7.4	-7.9	-7.3	-7.8	-6.1	-9.1	-7.6	-7.1	-8.8	-7.6	-7.7	-8.0	-8.3	-7.0	-6.8	-4.4	train	train	train	val	test	train	train
123	CHEMBL420053	low	nh	nhm	a	-7.0	-6.8	-6.9	-6.3	-7.2	-6.0	-7.6	-7.4	-6.3	-7.9	-7.3	-6.8	-7.9	-7.8	-6.2	-5.8	-4.6	train	train	train	train	val	test	train
124	CHEMBL205164	low	nh	nhm	a	-6.7	-7.1	-7.4	-5.1	-7.5	-6.4	-7.6	-7.0	-6.8	-8.0	-7.6	-6.9	-7.5	-7.2	-6.3	-5.8	-4.2	train	train	train	train	train	val	test
125	CHEMBL204131	low	nh	nhm	a	-7.9	-7.4	-8.2	-5.6	-7.7	-6.5	-8.9	-7.6	-6.8	-8.8	-7.4	-7.4	-8.5	-7.8	-6.9	-6.5	-3.8	test	train	train	train	train	train	val
126	CHEMBL203651	low	nh	nhm	a	-7.8	-8.0	-8.0	-5.7	-8.0	-6.7	-9.2	-8.6	-6.9	-8.7	-7.8	-7.8	-8.2	-7.9	-6.8	-6.7	-4.8	val	test	train	train	train	train	train
127	CHEMBL382775	low	nh	nhm	a	-7.2	-7.2	-7.7	-5.6	-7.7	-6.5	-8.5	-7.8	-6.7	-7.7	-7.6	-7.4	-7.8	-7.4	-6.6	-6.3	-4.1	train	val	test	train	train	train	train
128	CHEMBL203968	low	nh	nhm	a	-7.3	-7.1	-7.8	-5.4	-8.0	-6.6	-8.2	-7.6	-7.0	-8.7	-7.3	-7.6	-7.6	-8.1	-6.8	-6.6	-4.2	train	train	val	test	train	train	train
129	CHEMBL4078967	low	nh	nhm	a	-8.1	-9.5	-9.4	-6.7	-9.4	-7.9	-9.5	-8.9	-8.2	-10.1	-8.8	-7.0	-7.5	-7.9	-7.6	-7.0	-4.5	train	train	train	val	test	train	train
130	CHEMBL156834	low	nh	nhm	a	-7.8	-7.2	-8.1	-7.9	-7.7	-6.2	-8.6	-7.6	-6.8	-8.0	-7.5	-8.3	-8.2	-8.4	-7.1	-7.1	-5.5	train	train	train	train	val	test	train

131	CHEMBL206705	low	nh	nhm	a	-8.1	-7.5	-8.4	-5.7	-8.5	-6.8	-9.1	-7.8	-6.8	-9.1	-7.6	-7.7	-8.8	-8.8	-7.3	-7.1	-4.6	train	train	train	train	train	val	test
132	CHEMBL380524	low	nh	nhm	a	-8.1	-7.3	-8.2	-6.5	-7.4	-6.5	-9.0	-7.4	-6.8	-8.6	-7.5	-8.0	-8.7	-7.9	-6.9	-6.6	-4.2	test	train	train	train	train	train	val
133	CHEMBL203833	low	nh	nhm	a	-7.8	-7.0	-8.3	-7.4	-8.3	-6.5	-9.3	-8.1	-6.7	-8.4	-7.6	-8.1	-8.4	-8.5	-7.1	-6.6	-4.6	val	test	train	train	train	train	train
134	CHEMBL155571	low	nh	nhm	a	-7.8	-7.8	-8.4	-7.7	-8.0	-6.8	-8.9	-7.9	-6.8	-8.4	-8.0	-8.8	-8.4	-9.0	-7.5	-7.3	-5.8	train	val	test	train	train	train	train
135	CHEMBL204553	low	nh	nhm	a	-8.2	-6.8	-7.8	-5.2	-7.4	-6.1	-7.9	-7.7	-6.5	-8.6	-7.8	-7.6	-8.4	-8.5	-7.0	-6.4	-4.0	train	train	val	test	train	train	train
136	CHEMBL85348	low	nh	nhm	a	-6.4	-7.4	-6.9	-6.9	-7.2	-6.1	-7.6	-7.4	-6.2	-8.3	-7.4	-7.0	-8.4	-8.2	-6.7	-6.0	-4.8	train	train	train	val	test	train	train
137	CHEMBL206037	low	nh	nhm	a	-8.1	-7.1	-7.6	-5.3	-7.8	-6.2	-8.7	-7.6	-7.0	-8.1	-7.9	-7.6	-8.1	-7.8	-6.8	-6.4	-4.1	train	train	train	train	val	test	train
138	CHEMBL156241	low	nh	nhm	a	-8.1	-7.3	-8.8	-9.1	-8.8	-7.0	-9.2	-8.4	-7.3	-9.2	-8.7	-9.1	-8.9	-9.5	-7.6	-7.7	-6.1	train	train	train	train	train	val	test
139	CHEMBL204205	low	nh	nhm	a	-7.9	-6.8	-7.8	-5.5	-8.0	-6.1	-8.5	-7.7	-6.3	-8.4	-7.5	-7.5	-8.8	-8.4	-6.8	-6.7	-4.2	test	train	train	train	train	train	val
140	CHEMBL315852	low	nh	nhm	a	-6.1	-6.1	-6.9	-6.2	-7.0	-5.5	-7.3	-7.0	-6.2	-7.4	-7.1	-7.0	-7.6	-7.7	-5.9	-5.3	-4.2	val	test	train	train	train	train	train
141	CHEMBL203217	low	nh	nhm	a	-7.4	-7.1	-7.5	-5.2	-7.7	-6.4	-7.5	-7.7	-6.8	-8.0	-7.8	-7.3	-8.2	-7.7	-6.2	-6.1	-4.2	train	val	test	train	train	train	train
142	CHEMBL206338	low	nh	nhm	a	-7.6	-7.2	-7.8	-6.3	-7.4	-6.1	-9.2	-7.9	-6.7	-8.5	-8.0	-7.8	-8.7	-9.0	-6.9	-6.8	-3.9	train	train	val	test	train	train	train
143	CHEMBL381174	low	nh	nhm	a	-7.9	-7.3	-8.1	-5.8	-7.9	-6.5	-8.7	-7.7	-6.7	-8.6	-7.9	-7.8	-8.6	-8.4	-7.0	-6.5	-4.4	train	train	train	val	test	train	train
144	CHEMBL315703	low	nh	nhm	a	-7.1	-7.3	-7.0	-6.5	-7.3	-6.4	-7.7	-7.4	-6.4	-8.0	-7.6	-7.5	-8.0	-7.8	-6.7	-5.9	-4.5	train	train	train	train	val	test	train
145	CHEMBL382485	low	nh	nhm	a	-7.6	-7.0	-7.8	-6.4	-7.2	-6.3	-8.6	-7.3	-6.7	-8.2	-7.9	-7.6	-8.4	-7.8	-6.6	-6.3	-4.0	train	train	train	train	train	val	test
146	CHEMBL203572	low	nh	nhm	a	-7.7	-7.1	-7.7	-5.3	-7.4	-6.4	-8.6	-7.7	-6.8	-8.4	-7.6	-7.7	-8.4	-8.0	-6.7	-6.4	-4.2	test	train	train	train	train	train	val
147	CHEMBL204307	low	nh	nhm	a	-7.8	-8.1	-8.4	-6.0	-8.5	-6.7	-8.2	-8.5	-7.3	-9.3	-8.1	-8.2	-8.6	-7.8	-7.5	-7.1	-4.8	val	test	train	train	train	train	train
148	CHEMBL381072	low	nh	nhm	a	-7.0	-6.5	-6.8	-6.1	-7.3	-5.4	-7.8	-6.8	-6.1	-7.4	-7.0	-7.2	-7.7	-7.9	-6.1	-6.0	-3.5	train	val	test	train	train	train	train
149	CHEMBL381915	low	nh	nhm	a	-7.8	-7.4	-8.2	-5.5	-7.7	-6.5	-9.0	-7.6	-6.8	-8.8	-7.7	-7.0	-8.5	-7.8	-7.0	-6.5	-3.8	train	train	val	test	train	train	train
150	CHEMBL204855	low	nh	nhm	a	-7.4	-7.1	-7.7	-7.2	-7.5	-6.2	-9.1	-8.1	-6.8	-8.5	-8.1	-7.5	-8.4	-7.8	-6.7	-6.7	-4.7	train	train	train	val	test	train	train
151	CHEMBL431022	low	nh	nhm	a	-7.0	-6.9	-7.0	-6.6	-7.2	-6.2	-7.5	-7.4	-6.2	-7.8	-7.3	-7.3	-8.1	-7.9	-6.3	-6.1	-4.3	train	train	train	train	val	test	train
152	CHEMBL4080811	low	nh	nhm	a	-8.0	-9.7	-9.6	-6.6	-9.2	-8.3	-10.3	-10.0	-8.7	-10.0	-9.7	-9.3	-11.1	-11.3	-8.1	-8.8	-5.4	train	train	train	train	train	val	test
153	CHEMBL4088680	low	nh	nhm	a	-7.8	-9.7	-9.7	-6.8	-9.7	-7.9	-10.0	-9.8	-9.7	-10.3	-10.5	-9.9	-10.4	-10.9	-8.4	-7.9	-5.6	test	train	train	train	train	train	val
154	CHEMBL4095476	low	nh	nhm	a	-7.4	-9.3	-9.2	-6.7	-9.1	-8.5	-8.7	-8.6	-8.3	-10.3	-9.4	-7.3	-9.1	-8.2	-8.2	-7.5	-4.1	val	test	train	train	train	train	train
155	CHEMBL85707	low	nh	nhm	a	-7.2	-7.5	-8.4	-5.7	-8.0	-6.8	-8.4	-8.1	-7.2	-8.9	-8.3	-8.0	-8.3	-7.8	-6.9	-6.8	-4.7	train	val	test	train	train	train	train
156	CHEMBL382483	low	nh	nhm	a	-7.4	-7.3	-8.1	-6.2	-7.7	-6.4	-8.8	-7.6	-7.1	-8.5	-8.2	-8.0	-8.7	-7.9	-6.7	-6.5	-4.1	train	train	val	test	train	train	train
157	CHEMBL3246756	low	nh	nhm	a	-7.3	-8.7	-8.3	-6.9	-8.9	-7.1	-10.2	-8.9	-7.6	-9.5	-9.1	-9.5	-9.4	-9.1	-7.6	-7.1	-5.1	train	train	train	val	test	train	train
158	CHEMBL3277541	low	nh	nhm	a	-8.1	-8.1	-8.3	-5.9	-8.3	-7.5	-9.1	-8.4	-7.7	-9.0	-8.6	-8.8	-9.3	-10.0	-7.1	-7.4	-4.3	train	train	train	train	val	test	train
159	CHEMBL1949977	low	nh	nhm	a	-7.7	-8.7	-9.7	-7.0	-9.8	-7.9	-11.0	-10.3	-9.6	-11.1	-9.6	-7.3	-8.7	-7.6	-8.4	-8.8	-4.7	train	train	train	train	train	val	test
160	CHEMBL449929	low	nh	nhm	a	-7.8	-7.4	-8.0	-6.2	-8.1	-6.7	-8.5	-7.5	-7.0	-8.6	-8.6	-7.5	-8.2	-7.4	-7.5	-7.0	-4.7	test	train	train	train	train	train	val
161	CHEMBL3360996	low	nh	nhm	a	-7.8	-8.3	-9.2	-6.3	-8.7	-7.3	-9.3	-9.2	-8.2	-9.6	-9.4	-7.9	-8.0	-7.0	-7.5	-7.6	-4.7	val	test	train	train	train	train	train
162	CHEMBL3763633	active	nh	nhm	a	-7.8	-7.3	-8.4	-5.8	-8.3	-6.7	-8.5	-8.4	-7.3	-9.2	-8.2	-7.9	-8.2	-8.3	-7.0	-7.2	-4.6	train	val	test	train	train	train	train
163	CHEMBL66654	active	nh	nhm	a	-7.8	-6.9	-7.5	-7.3	-7.2	-5.8	-8.4	-7.2	-6.4	-8.5	-7.2	-8.0	-7.8	-8.5	-7.0	-7.2	-4.4	train	train	val	test	train	train	train
164	CHEMBL2181582	active	nh	nhm	a	-9.0	-8.9	-9.1	-6.8	-9.3	-8.1	-10.0	-9.7	-7.8	-10.2	-9.3	-8.8	-9.2	-10.1	-8.1	-7.5	-5.8	train	train	train	val	test	train	train
165	CHEMBL3356152	active	nh	nhm	a	-6.9	-7.2	-8.0	-6.0	-7.9	-6.7	-8.1	-8.1	-6.5	-8.5	-8.3	-8.0	-7.8	-8.1	-6.7	-6.5	-4.3	train	train	train	train	val	test	train
166	CHEMBL1828889	active	nh	nhm	a	-7.4	-7.7	-7.4	-5.2	-7.6	-6.5	-8.9	-7.6	-6.7	-8.3	-7.9	-8.0	-8.3	-8.3	-6.4	-6.3	-4.1	train	train	train	train	train	val	test
167	CHEMBL3356151	active	nh	nhm	a	-7.4	-7.3	-7.9	-7.4	-8.2	-6.6	-8.2	-7.9	-7.3	-7.9	-7.6	-8.1	-8.1	-8.2	-6.6	-6.4	-4.6	test	train	train	train	train	train	val
168	CHEMBL3764390	active	nh	nhm	a	-7.8	-8.0	-8.5	-6.3	-8.7	-7.0	-8.7	-8.6	-7.8	-9.7	-8.5	-8.6	-8.1	-8.6	-7.3	-7.3	-4.7	val	test	train	train	train	train	train
169	CHEMBL3356148	active	nh	nhm	a	-6.8	-7.0	-7.8	-5.9	-8.2	-6.5	-8.1	-7.7	-6.7	-8.0	-7.9	-7.9	-8.1	-8.2	-6.8	-6.5	-4.0	train	val	test	train	train	train	train
170	CHEMBL256339	active	nh	nhm	a	-6.5	-6.1	-7.0	-5.9	-7.0	-5.7	-7.4	-6.7	-5.8	-7.1	-6.2	-6.8	-7.0	-7.5	-6.3	-5.7	-4.5	train	train	val	test	train	train	train
171	CHEMBL3246238	active	nh	nhm	a	-7.0	-7.5	-7.6	-7.6	-7.9	-6.1	-9.2	-7.8	-7.3	-8.7	-7.9	-7.9	-8.6	-8.3	-7.0	-7.0	-4.4	train	train	train	val	test	train	train
172	CHEMBL45	active	nh	nhm	a	-7.4	-6.6	-7.7	-8.0	-7.5	-6.0	-8.3	-7.4	-6.3	-7.4	-7.2	-8.0	-7.3	-7.8	-6.3	-6.1	-5.9	train	train	train	train	val	test	train
173	CHEMBL539698	active	nh	nhm	a	-7.8	-7.8	-8.2	-5.8	-8.1	-7.2	-8.6	-8.6	-7.3	-8.9	-8.2	-7.0	-8.2	-8.3	-6.7	-6.5	-5.0	train	train	train	train	train	val	test
174	CHEMBL1087164	active	nh	nhm	a	-8.1	-7.3	-8.4	-5.6	-9.6	-6.6	-7.9	-7.7	-7.7	-9.3	-8.5	-6.9	-6.5	-7.0	-6.7	-7.4	-4.4	test	train	train	train	train	train	val
175	CHEMBL235327	active	nh	nhm	a	-7.4	-7.6	-7.7	-8.2	-7.6	-6.6	-8.4	-7.4	-6.5	-9.0	-7.8	-7.9	-8.8	-8.7	-6.6	-6.4	-3.9	val	test	train	train	train	train	train
176	CHEMBL391604	active	nh	nhm	a	-6.4	-6.4	-7.0	-5.2	-6.2	-5.8	-7.5	-6.7	-6.0	-7.3	-6.8	-7.4	-7.1	-6.7	-6.5	-5.1	-3.7	train	val	test	train	train	train	train
177	CHEMBL390950	active	nh	nhm	a	-7.3	-7.5	-7.8	-7.2	-8.1	-6.5	-8.1	-8.2	-6.9	-8.6	-7.9	-7.4	-8.0	-8.4	-6.4	-6.8	-4.9	train	train	val	test	train	train	train
178	CHEMBL3600401	active	nh	nhm	a	-7.8	-7.9	-8.6	-5.8	-8.0	-6.7	-8.8	-8.6	-7.7	-8.7	-8.4	-7.5	-8.1	-7.6	-7.9	-7.2	2.3	train	train	train	val	test	train	train
179	CHEMBL206296	active	nh	nhm	a	-7.4	-6.9	-7.7	-7.6	-7.6	-6.2	-8.0	-7.5	-6.7	-7.9	-7.6	-7.8	-7.6	-8.1	-6.4	-6.2	-4.5	train	train	train	train	val	test	train
180	CHEMBL2179195	active	nh	nhm	a	-5.6	-7.1	-6.9	-4.7	-7.9	-6.0	-7.2	-7.9	-7.1	-8.8	-7.5	-6.1	-5.0	-5.8	-6.4	-6.1	7.0	train	train	train	train	train	val	test
181	CHEMBL4208744	active	nh	nhm	a	-8.4	-8.4	-8.6	-6.3	-8.6	-8.5	-8.8	-9.1	-7.5	-9.4	-8.1	-8.0	-8.2	-8.5	-7.1	-7.1	-4.8	test	train	train	train	train	train	val
182	CHEMBL127135	active	nh	nhm	a	-8.8	-8.1	-9.5	-7.9	-9.1	-7.2	-9.9	-9.1	-7.8	-9.7	-8.2	-9.1	-9.7	-9.5	-8.0	-7.6	-5.5	val	test	train	train	train	train	train
183	CHEMBL2440659	active	nh	nhm	a	-7.7	-8.1	-8.6	-6.5	-7.9	-6.9	-9.1	-8.4	-7.8	-8.8	-7.8	-8.8	-7.9	-8.9	-7.3	-7.1	-4.6	train	val	test	train	train	train	train
184	CHEMBL1275998	active	nh	nhm	a	-7.7	-7.4	-7.9	-7.3	-8.8	-6.1	-8.8	-7.8	-7.4	-8.3	-7.9	-8.0	-8.5	-9.3	-6.5	-6.6	-4.4	train	train	val	test	train	train	train
185	CHEMBL3356150	active	nh	nhm	a	-7.3	-7.2	-7.8	-6.3	-8.2	-6.6	-8.1	-7.8	-6.9	-8.2	-7.6	-8.2	-8.2	-8.1	-6.6	-6.4	-4.3	train	train	train	val	test	train	train
186	CHEMBL239800	active	nh	nhm	a	-7.4	-7.2	-7.6	-6.9	-7.3	-6.4	-8.9	-7.2	-6.5	-7.9	-7.7	-7.7	-7.7	-8.2	-6.6	-5.9	-4.2	train	train	train	train	val	test	train
187	CHEMBL292065	active	nh	nhm	a	-6.7	-6.2	-6.3	-5.9	-6.4	-5.1	-7.3	-6.1	-5.8	-6.9	-6.2	-6.8	-6.6	-6.8	-5.9	-5.6	-3.6	train	train	train	train	train	val	test
188	CHEMBL2181572	active	nh	nhm	a	-8.9	-8.4	-9.0	-6.6	-9.1	-6.9	-9.3	-9.8	-7.8	-10.2	-9.1	-7.8	-8.3	-9.4	-7.9	-7.4	-5.8	test	train	train	train	train	train	val
189	CHEMBL2181576	active	nh	nhm	a	-8.5	-8.6	-8.7	-6.3	-9.1	-7.1	-9.5	-9.3	-8.0	-10.0	-9.2	-8.3	-7.8	-9.7	-7.8	-7.7	-5.5	val	test	train	train	train	train	train
190	CHEMBL104593	active	nh	nhm	a	-7.8	-7.3	-8.4	-5.8	-8.5	-6.4	-8.8	-8.6	-7.1	-8.4	-8.2	-7.6	-8.1	-8.2	-7.0	-6.7	-4.6	train	val	test	train	train	train	train
191	CHEMBL2181578	active	nh	nhm	a	-9.0	-8.8	-9.3	-6.8	-9.4	-7.9	-9.9	-9.8	-7.7	-10.1	-9.2	-9.0	-9.3	-10.1	-8.0	-7.4	-5.8	train	train	val	test	train	train	train
192	CHEMBL4209954	active	nh	nhm	a	-7.5	-8.1	-7.7	-6.3	-7.7	-7.6	-8.4	-8.3	-7.2	-8.6	-7.9	-7.4	-7.5	-8.1	-6.9	-6.5	-4.4	train	train	train	val	test	train	train
193	CHEMBL282199	active	nh	nhm	a	-8.3	-7.6	-7.6	-6.2	-8.4	-6.9	-9.1	-8.9	-7.5	-8.7	-8.5	-8.1	-8.2	-9.1	-6.9	-6.8	-4.6	train	train	train	train	val	test	train
194	CHEMBL3360999	active	nh	nhm	a	-8.0	-8.2	-9.1	-6.3	-8.6	-7.2	-9.0	-9.0	-8.1	-10.3	-9.1	-7.9	-8.0	-7.1	-7.6	-7.4	-5.0	train	train	train	train	train	val	test
195	CHEMBL560437	active	nh	nhm	a	-9.0	-8.4	-9.2	-6.4	-9.1	-7.8	-9.5	-9.8	-7.7	-10.2	-9.1	-7.8	-8.7	-9.4	-7.6	-7.4	-5.8	test	train	train	train	train	train	val
196	CHEMBL85770	inactive	nh	nhm	na	-6.5	-6.7	-7.1	-6.9	-7.5	-6.1	-7.8	-7.5	-6.4	-8.0	-7.8	-6.9	-8.1	-8.2	-6.6	-6.3	-4.4	val	test	train	train	train	train	train

197	CHEMBL434	inactive	nh	nhm	na	-6.8	-5.9	-6.9	-7.7	-6.5	-5.5	-7.2	-6.2	-5.6	-6.9	-6.3	-7.0	-6.7	-7.0	-6.2	-5.4	-5.3	train	val	test	train	train	train	train
198	CHEMBL347242	inactive	nh	nhm	na	-8.6	-8.5	-9.8	-7.8	-9.1	-7.0	-10.2	-9.4	-8.0	-10.3	-9.1	-9.9	-10.4	-10.2	-8.3	-8.4	-5.1	train	train	val	test	train	train	train
199	CHEMBL156434	inactive	nh	nhm	na	-8.9	-8.4	-10.0	-9.7	-9.5	-7.4	-10.1	-9.8	-8.1	-10.1	-8.9	-10.2	-10.2	-10.3	-8.1	-8.4	-5.7	train	train	train	val	test	train	train
200	CHEMBL421154	inactive	nh	nhm	na	-6.2	-7.0	-7.2	-5.2	-7.3	-5.8	-8.1	-7.2	-6.6	-8.2	-7.4	-7.3	-8.0	-8.0	-6.6	-5.9	-4.6	train	train	train	train	val	test	train
201	CHEMBL312907	inactive	nh	nhm	na	-6.7	-6.7	-6.9	-7.0	-6.8	-5.9	-7.5	-7.2	-6.1	-7.4	-7.2	-6.9	-7.8	-7.7	-6.1	-5.7	-5.7	train	train	train	train	train	val	test
202	CHEMBL155168	inactive	nh	nhm	na	-7.6	-7.1	-8.2	-9.0	-8.0	-6.5	-8.9	-7.8	-6.8	-8.5	-7.8	-8.4	-8.4	-8.6	-7.2	-7.2	-5.6	test	train	train	train	train	train	val
203	CHEMBL153858	inactive	nh	nhm	na	-8.1	-7.2	-8.6	-8.0	-7.7	-6.4	-8.6	-7.9	-6.7	-8.4	-7.8	-8.8	-8.5	-8.8	-7.2	-7.4	-4.7	val	test	train	train	train	train	train
204	CHEMBL87530	inactive	nh	nhm	na	-6.4	-6.0	-6.7	-7.1	-6.8	-5.4	-7.4	-6.7	-5.9	-7.2	-6.8	-7.3	-6.9	-7.3	-6.0	-5.4	-4.0	train	val	test	train	train	train	train
205	CHEMBL87683	inactive	nh	nhm	na	-6.7	-6.2	-7.0	-7.4	-7.2	-5.5	-7.4	-6.7	-6.2	-6.9	-6.5	-7.3	-7.0	-7.0	-5.9	-5.9	-5.7	train	train	val	test	train	train	train
206	CHEMBL315700	inactive	nh	nhm	na	-5.8	-6.1	-6.6	-6.4	-6.7	-5.4	-7.4	-6.8	-6.1	-7.1	-7.0	-6.6	-6.9	-7.2	-6.0	-5.3	-3.8	train	train	train	val	test	train	train
207	CHEMBL155837	inactive	nh	nhm	na	-7.6	-7.2	-8.3	-9.1	-8.0	-6.6	-8.9	-8.0	-6.8	-8.6	-8.0	-8.5	-8.5	-8.8	-7.2	-7.3	-6.1	train	train	train	train	val	test	train
208	CHEMBL155598	inactive	nh	nhm	na	-7.7	-8.1	-9.4	-6.5	-8.3	-7.1	-9.7	-8.7	-7.2	-9.2	-8.8	-9.5	-9.5	-9.5	-8.2	-7.4	-6.5	train	train	train	train	train	val	test
209	CHEMBL85854	inactive	nh	nhm	na	-5.6	-6.3	-6.2	-5.6	-7.1	-5.4	-6.4	-6.9	-6.3	-6.9	-7.0	-6.0	-6.9	-7.2	-5.5	-5.5	-3.4	test	train	train	train	train	train	val
210	CHEMBL557688	inactive	nh	nhm	na	-8.4	-8.3	-8.6	-6.1	-8.5	-7.5	-9.0	-8.9	-7.5	-9.2	-8.5	-7.6	-8.4	-9.2	-7.2	-6.9	-5.2	val	test	train	train	train	train	train
211	CHEMBL87409	inactive	nh	nhm	na	-6.5	-6.4	-7.4	-8.0	-7.1	-5.7	-7.5	-7.3	-6.8	-7.3	-7.2	-7.7	-7.5	-7.5	-6.4	-5.9	-5.2	train	val	test	train	train	train	train
212	CHEMBL348532	inactive	nh	nhm	na	-7.9	-7.2	-8.3	-7.1	-7.8	-6.6	-8.8	-7.9	-6.7	-8.4	-8.2	-8.9	-8.4	-9.4	-7.3	-7.2	-4.7	train	train	val	test	train	train	train
213	CHEMBL2348441	inactive	nh	nhm	na	-8.6	-7.9	-8.9	-6.2	-8.6	-7.8	-9.2	-9.2	-7.7	-9.5	-8.7	-7.4	-8.2	-9.2	-7.5	-7.0	-5.3	train	train	train	val	test	train	train
214	CHEMBL2348446	inactive	nh	nhm	na	-8.4	-8.2	-8.6	-6.4	-8.9	-7.7	-9.5	-9.0	-7.5	-9.3	-8.7	-8.7	-8.7	-9.5	-7.6	-7.0	-5.3	train	train	train	train	val	test	train
215	CHEMBL1928323	inactive	nh	nhm	na	-6.6	-6.6	-6.9	-7.7	-6.8	-5.5	-8.3	-6.9	-6.1	-7.2	-7.0	-7.2	-7.4	-7.4	-5.9	-6.2	-3.9	train	train	train	train	train	val	test
216	CHEMBL4084733	inactive	nh	nhm	na	-9.4	-8.0	-9.6	-6.1	-9.2	-7.5	-10.1	-9.0	-7.6	-10.2	-8.6	-8.7	-10.0	-9.1	-8.2	-8.0	-4.9	test	train	train	train	train	train	val
