# Supplementary material for: Consensus Ensemble Multitarget Neural Network Model of Anxiolytic Activity of Chemical Compounds and Its Use for Multitarget Pharmacophore Design
Source: Pharmaceuticals (Basel). 2023 May 11;16(5):731. doi: 10.3390/ph16050731 (PMC10223244; doi:10.3390/ph16050731)
Supplement: Supplementary file 1 [file pharmaceuticals-16-00731-s001.zip › TableS8 DAB_RD Structures.pdf]

Table S8. Structural formulas of compounds with high anxiolytic activity and key points of their interaction with significant biotargets.

Record 1	
Structure	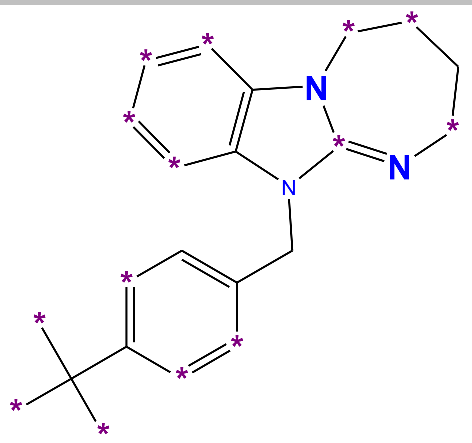
Code	DAB-0019
Target	ADRA1B
Record 2	
Structure	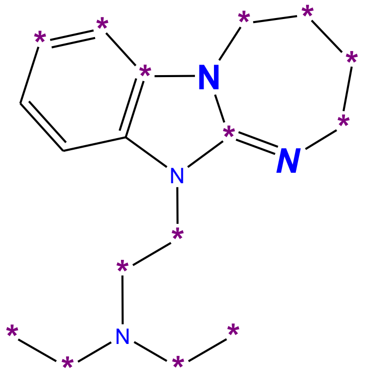
Code	DAB-0020
Target	ADRA1B
Record 3	
Structure	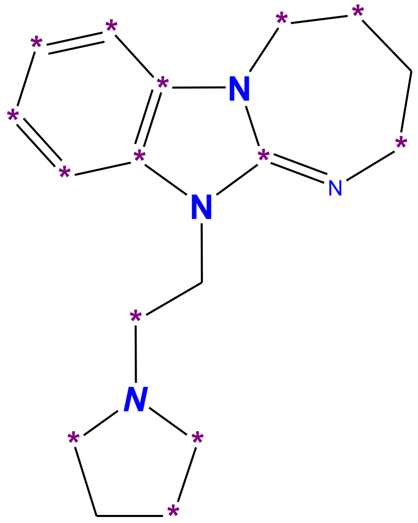
Code	DAB-0021
Target	ADRA1B
Record 4	

Structure

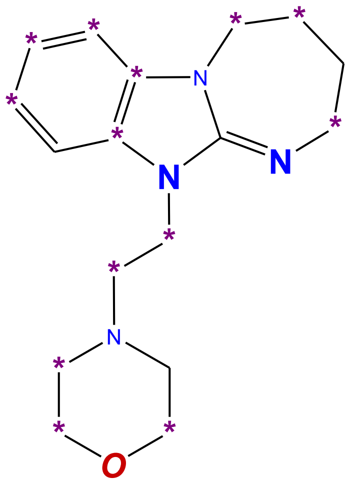

Code DAB-0023

Target ADRA1B

Record 5

Structure

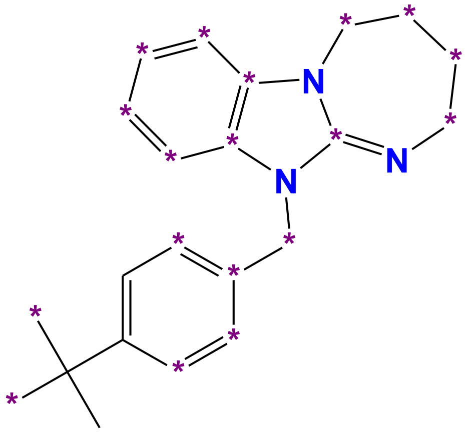

Code DAB-0019

Target ADRA2A

Record 6

Structure

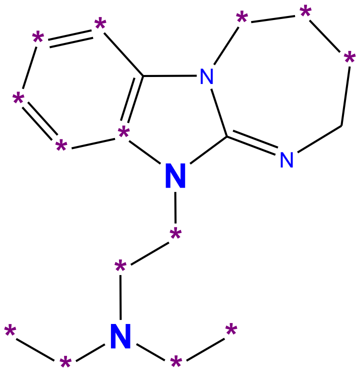

Code DAB-0020

Target ADRA2A

Record 7

Structure

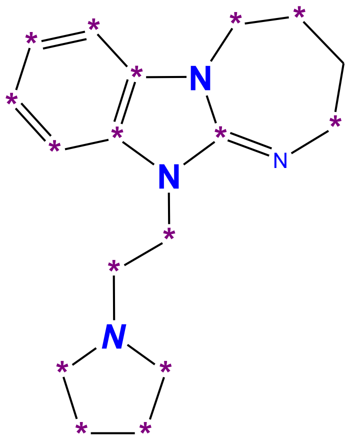

Code DAB-0021

Target ADRA2A

Record 8

Structure

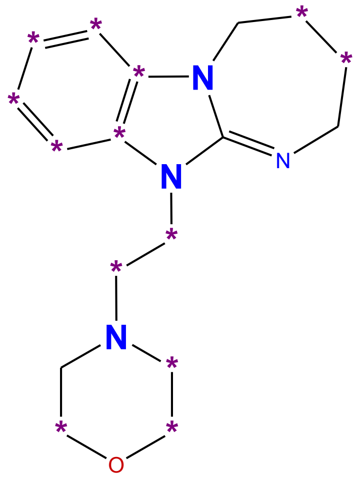

Code DAB-0023

Target ADRA2A

Record 9

Structure

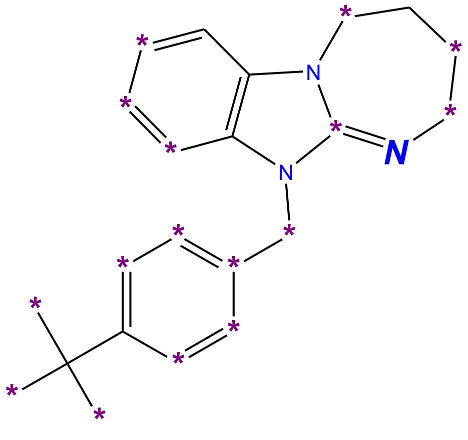

Code DAB-0019

Target AGTR1

Record 10

Structure

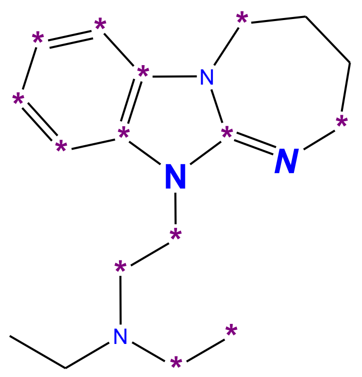

Code DAB-0020

Target AGTR1

Record 11

Structure

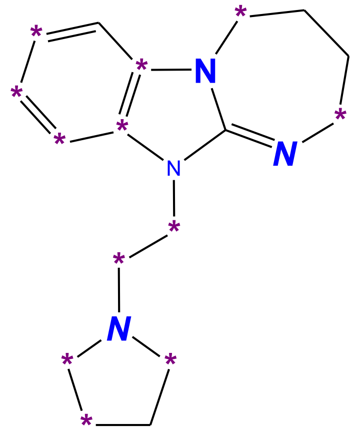

Code DAB-0021

Target AGTR1

Record 12

Structure

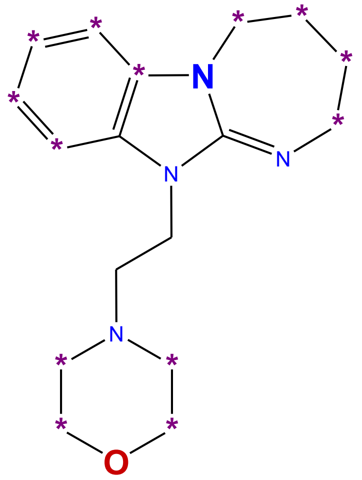

Code DAB-0023

Target AGTR1

Record 13

Structure

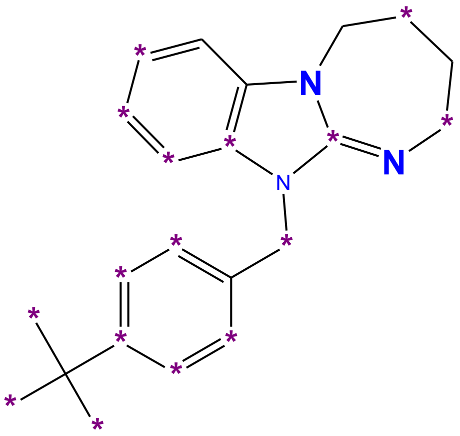

Code DAB-0019

Target NMDA-Glut

Record 14

Structure

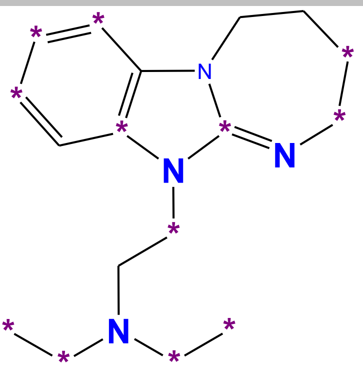

Code DAB-0020

Target NMDA-Glut

Record 15

Structure

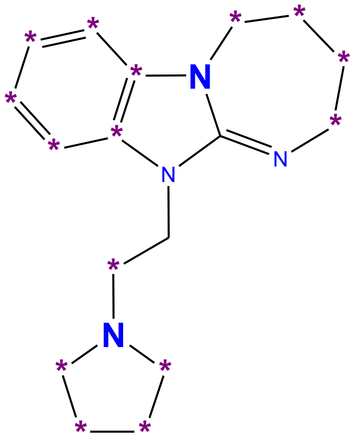

Code DAB-0021

Target NMDA-Glut

Record 16

Structure

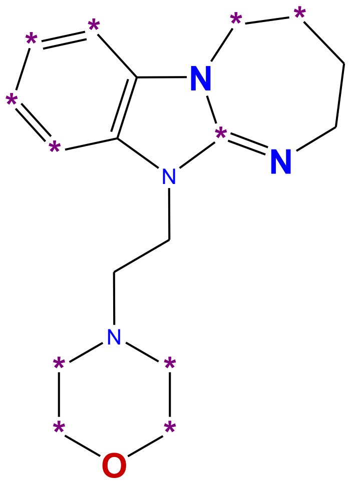

Code DAB-0023

Target NMDA-Glut

Record 17

Structure

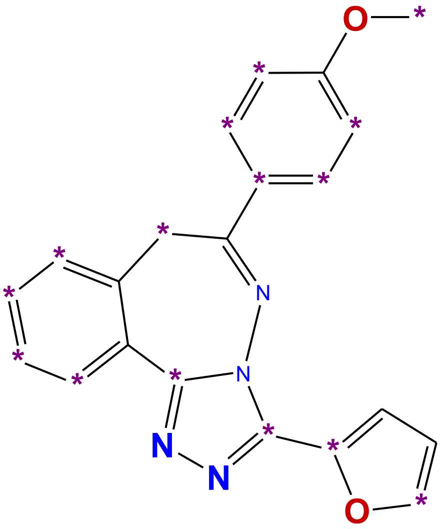

Code RD-0004

Target ADRA1B

Record 18

Structure

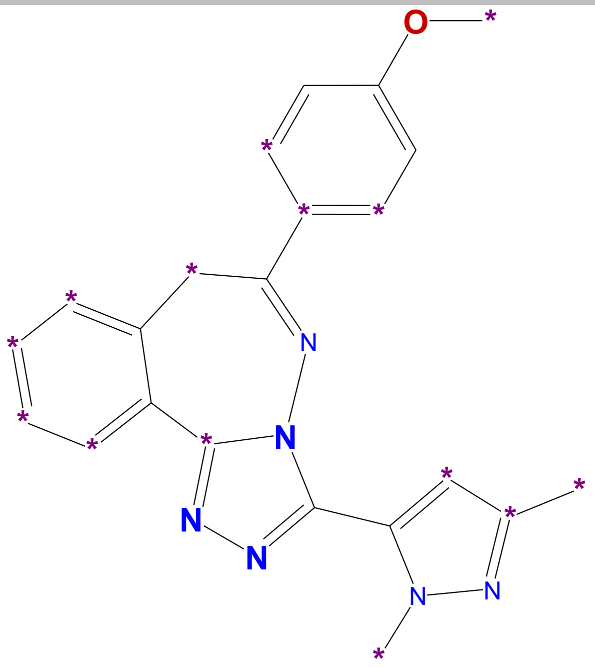

Code RD-0012

Target ADRA1B

Record 19

Structure

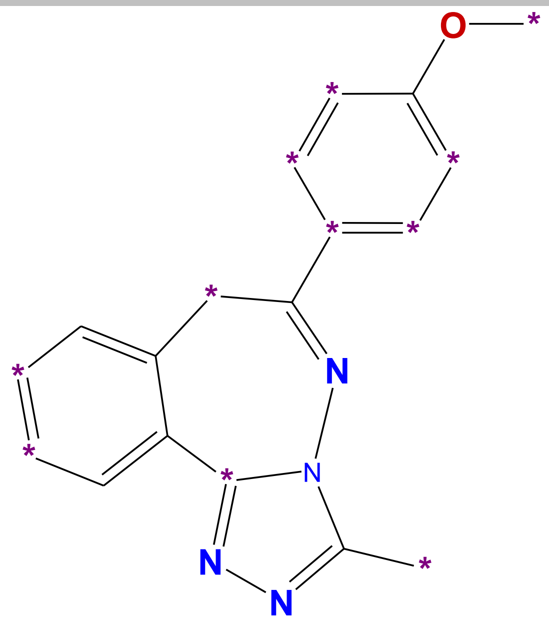

Code	RD-0013
Target	ADRA1B

Record 20

Structure

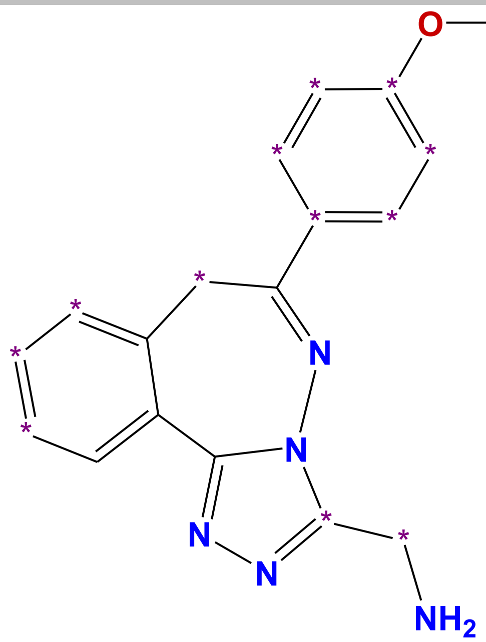

Code	RD-0014
Target	ADRA1B

Record 21

Structure

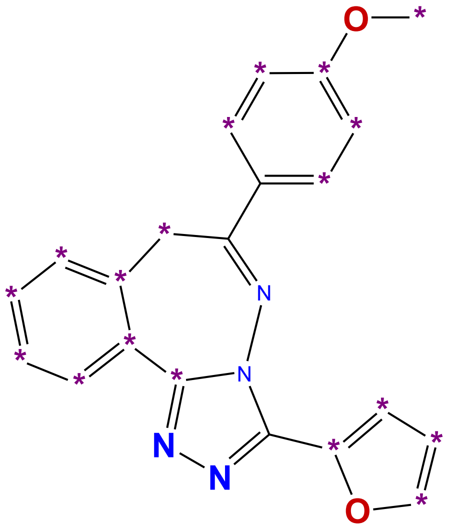

Code RD-0004

Target ADRA2A

Record 22

Structure

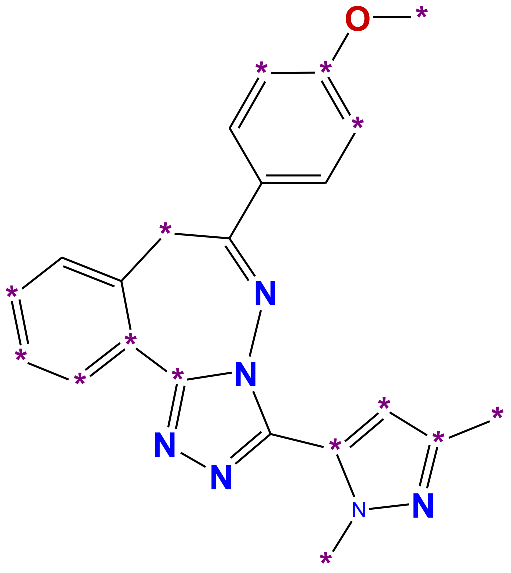

Code RD-0012

Target ADRA2A

Record 23

Structure

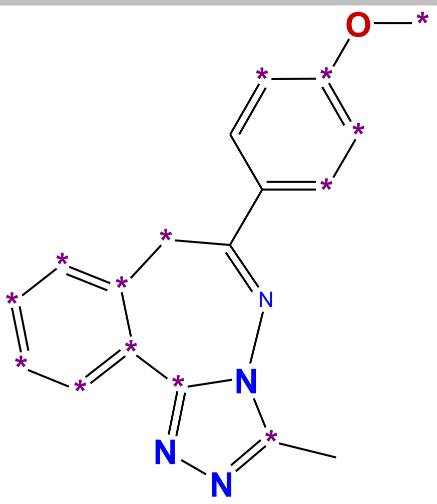

Code RD-0013

Target ADRA2A

Record 24

Structure

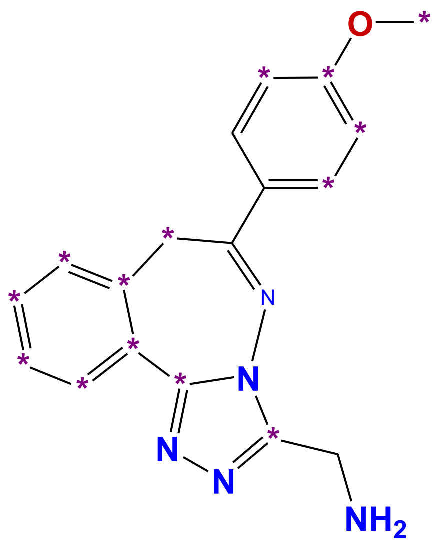

Code RD-0014

Target ADRA2A

Record 25

Structure

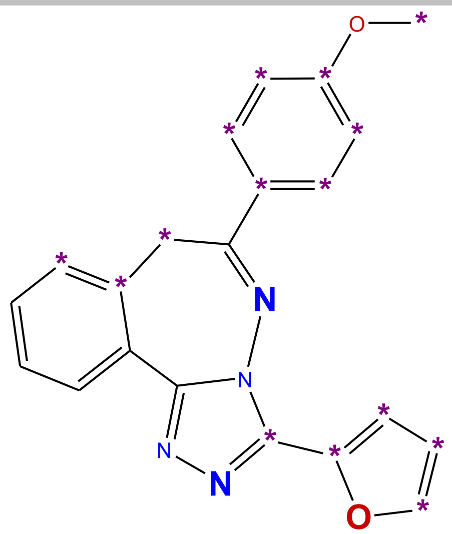

Code RD-0004

Target AGTR1

Record 26

Structure

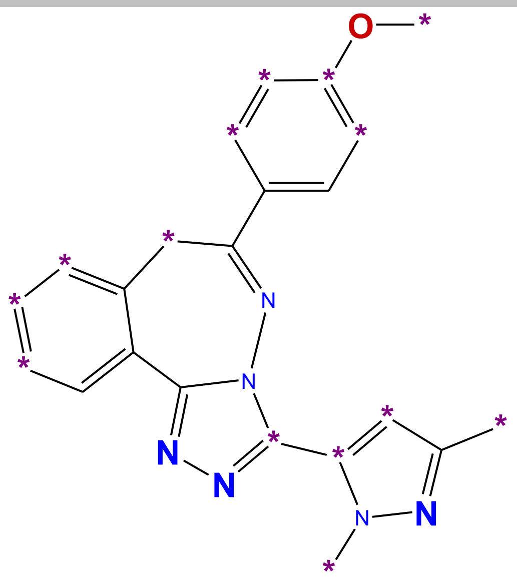

Code RD-0012

Target AGTR1

Record 27

Structure

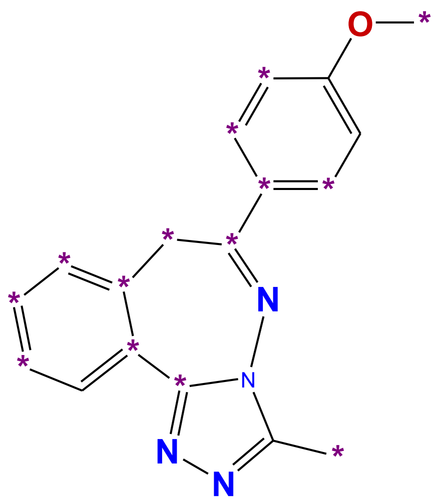

Code RD-0013

Target AGTR1

Record 28

Structure

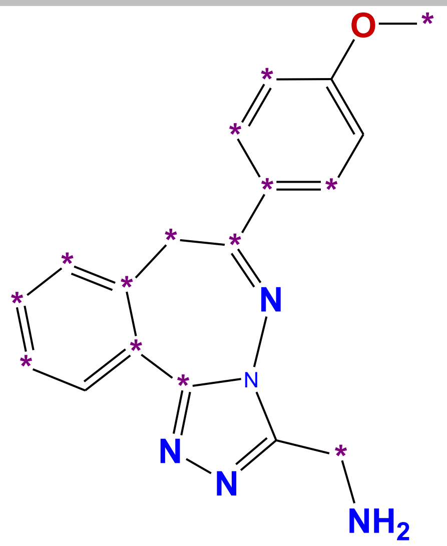

Code RD-0014

Target AGTR1

Record 29

Structure

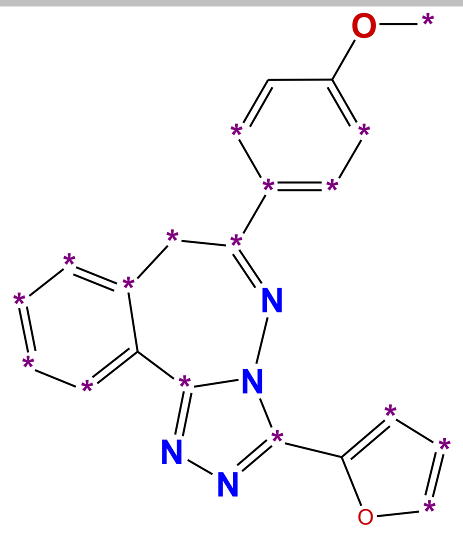

Code RD-0004

Target NMDA-Glut

Record 30

Structure

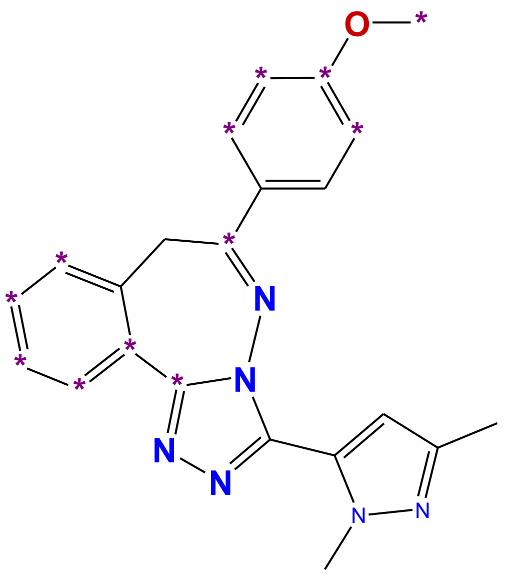

Code RD-0012

Target NMDA-Glut

Record 31

Structure

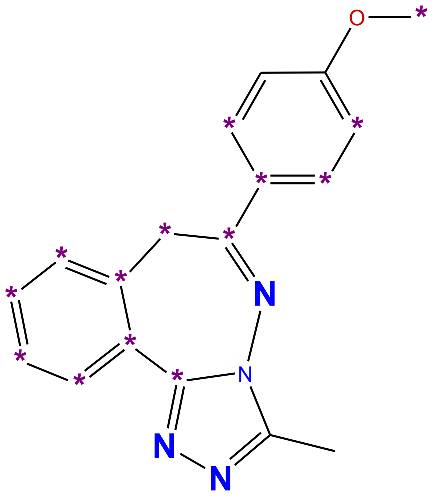

Code RD-0013

Target NMDA-Glut

Record 32

Structure

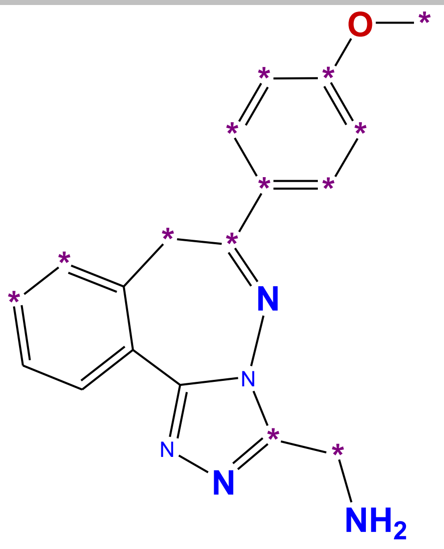

Code RD-0014

Target NMDA-Glut
