# Supplementary material for: Consensus Ensemble Multitarget Neural Network Model of Anxiolytic Activity of Chemical Compounds and Its Use for Multitarget Pharmacophore Design
Source: Pharmaceuticals (Basel). 2023 May 11;16(5):731. doi: 10.3390/ph16050731 (PMC10223244; doi:10.3390/ph16050731)
Supplement: Supplementary file 1 [file pharmaceuticals-16-00731-s001.zip › TableS9 Key Binding Amino Acids.pdf]

Table S9. Binding amino acids of sites of significant biotargets for novel compounds with anxiolytic activity.

11-substituted 2,3,4,5-tetrahydro-11H-[1,3]diazepino[1,2-a]benzimidazoles of the DAB series

Alpha-1B adrenergic receptor ADRA1B

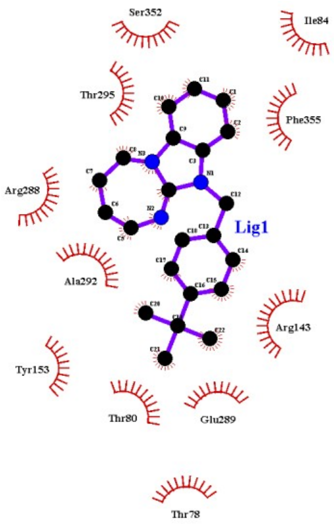

ADRA1B-DAB-0019

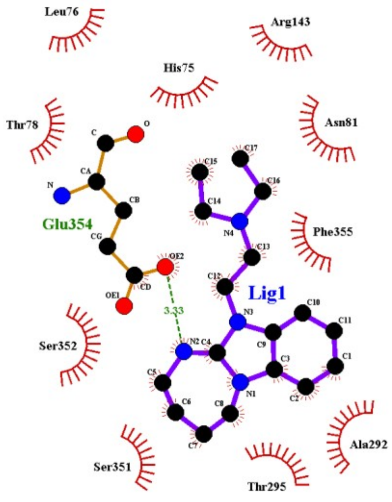

ADRA1B-DAB-0020

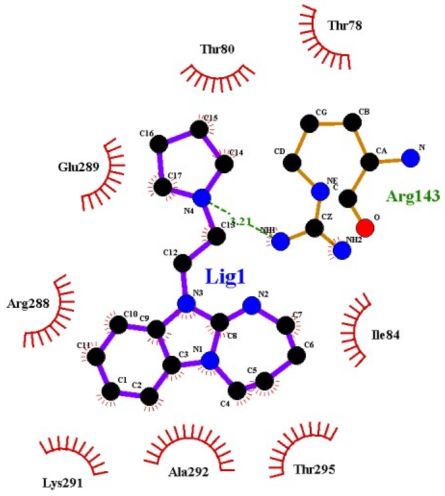

ADRA1B-DAB-0021

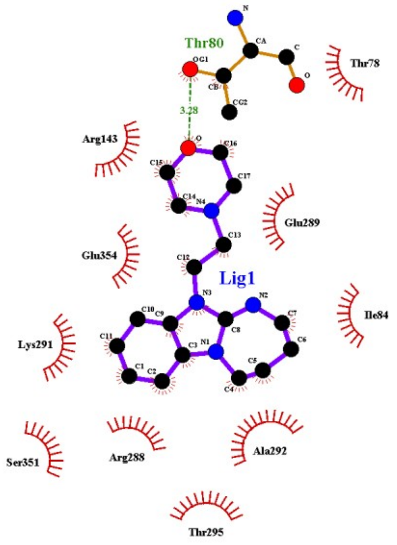

ADRA1B-DAB-0023

Alpha-2A adrenergic receptor ADRA2A

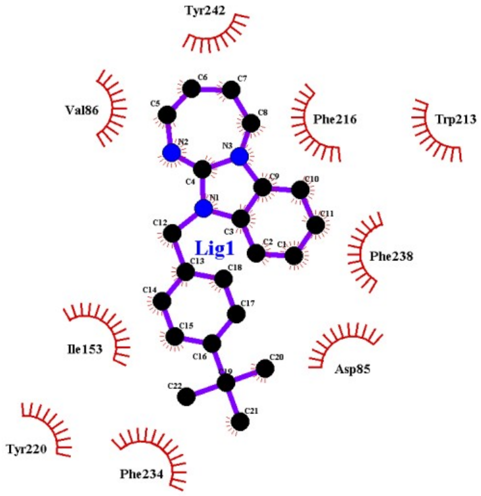

ADRA2A-DAB-0019

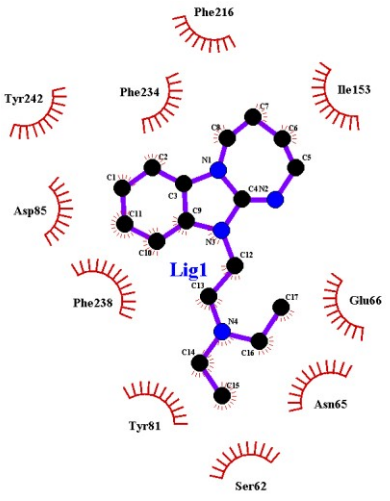

ADRA2A-DAB-0020

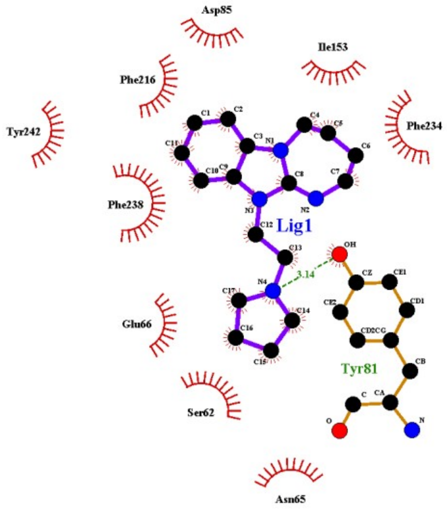

ADRA2A-DAB-0021

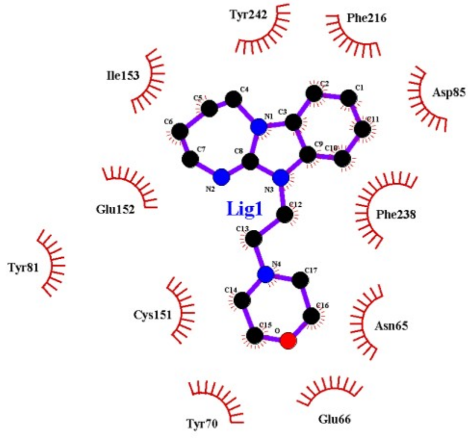

ADRA2A-DAB-0023

Type-1 angiotensin II receptor AGTR1

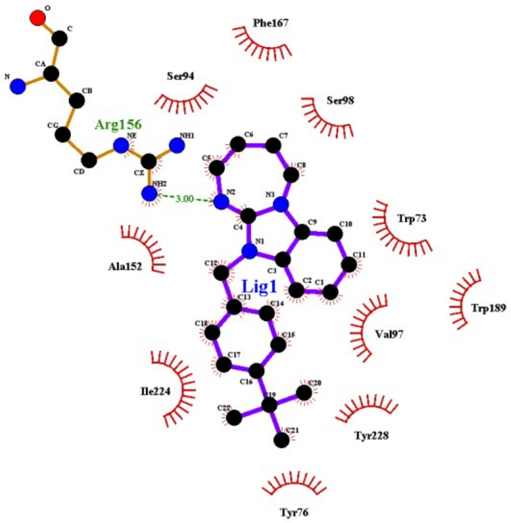

AGTR1-DAB-0019

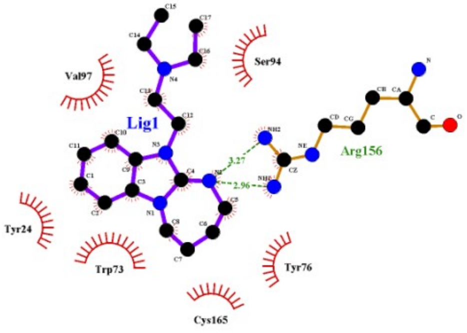

AGTR1-DAB-0020

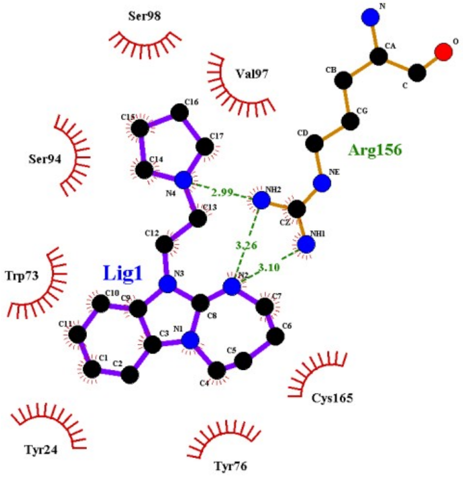

AGTR1-DAB-0021

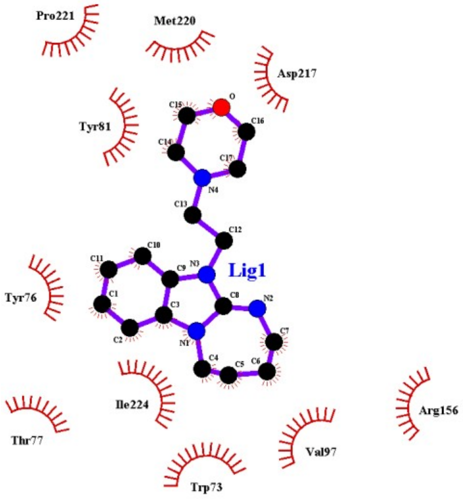

AGTR1-DAB-0023

NMDA receptor GluN1/GluN2A/GluN2B GRIN1/GRIN2A/GRIN2B

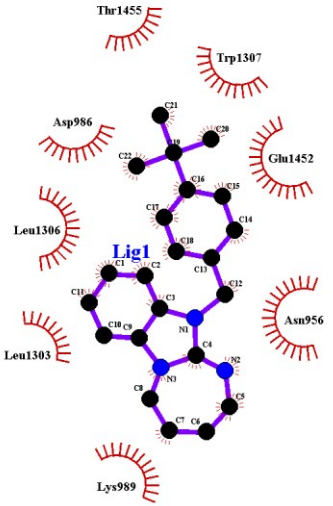

NMDA-Glut-DAB-0019

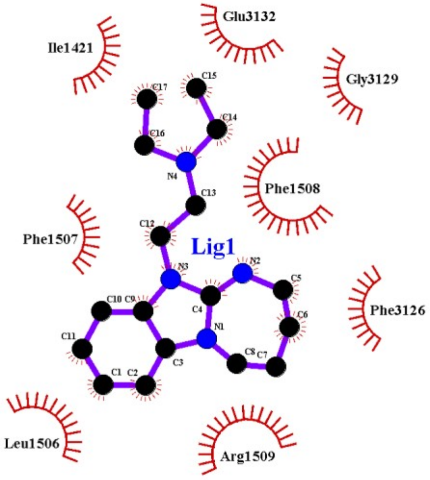

NMDA-Glut-DAB-0020

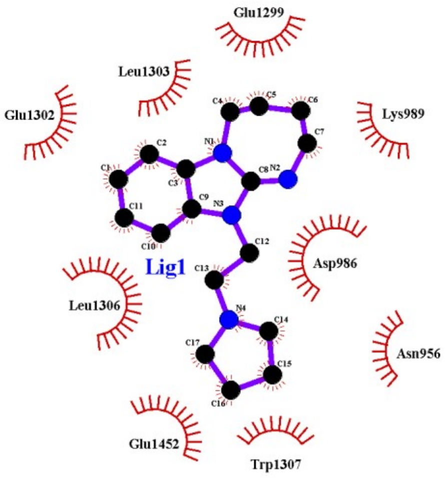

NMDA-Glut-DAB-0021

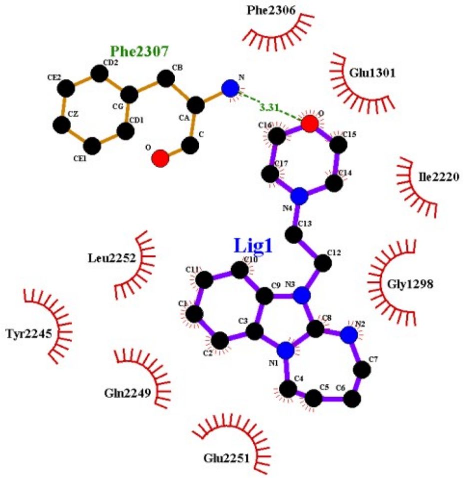

NMDA-Glut-DAB-0023

3,6-disubstituted [1,2,4]triazolo[3,4-a][2,3]benzodiazepines of the RD series

Alpha-1B adrenergic receptor ADRA1B

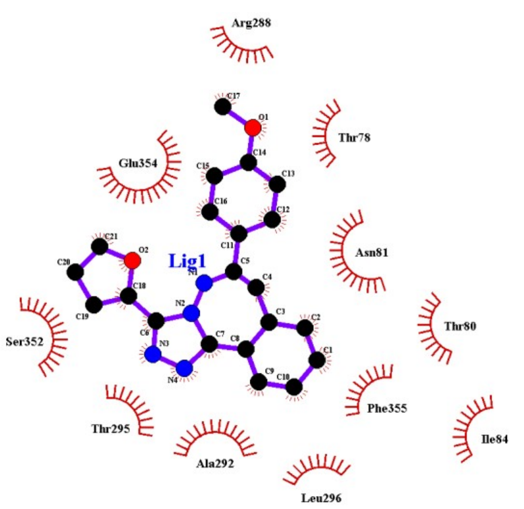

ADRA1B-RD-0004

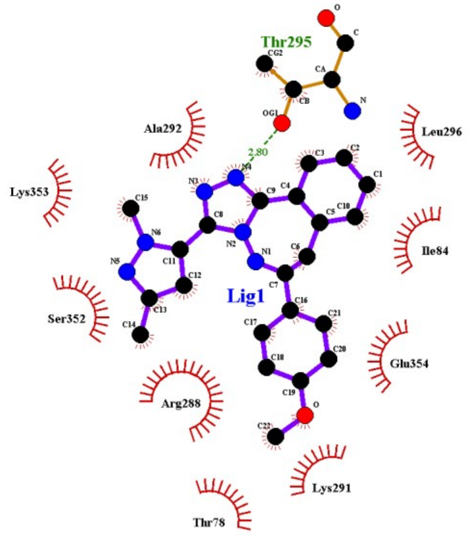

ADRA1B-RD-0012

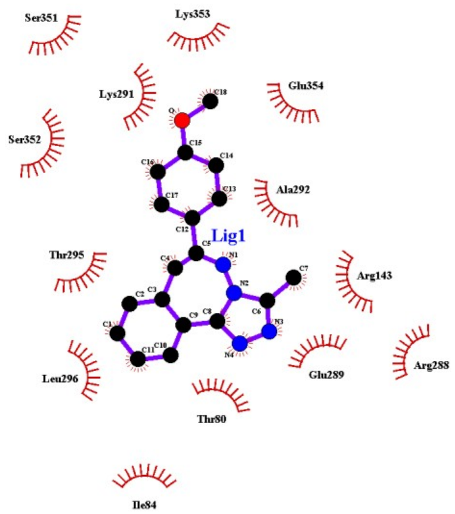

ADRA1B-RD-0013

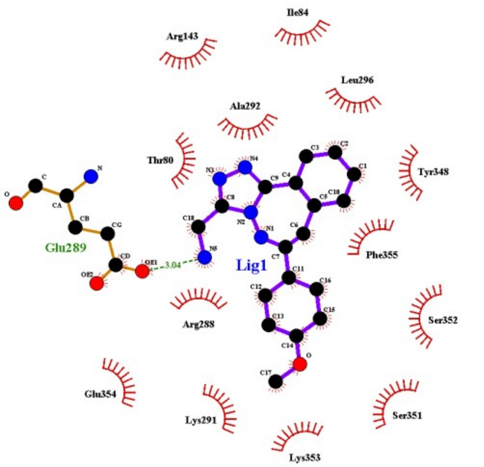

ADRA1B-RD-0014

Alpha-2A adrenergic receptor ADRA2A

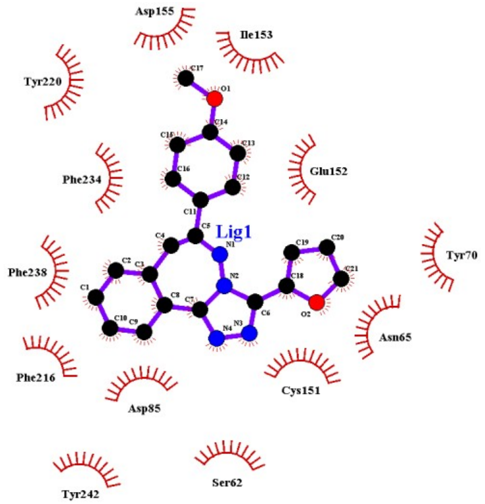

ADRA2A-RD-0004

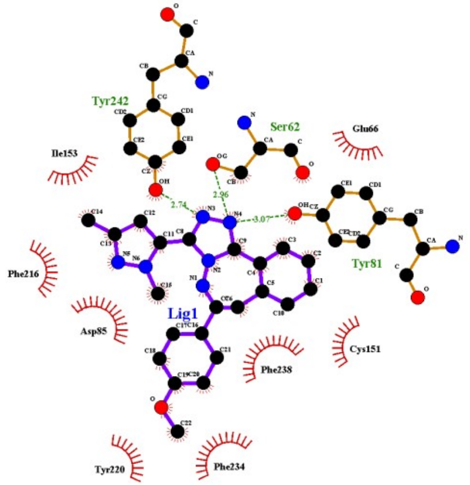

ADRA2A-RD-0012

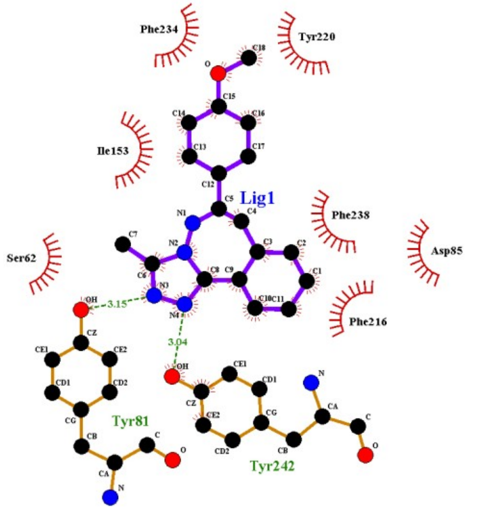

ADRA2A-RD-0013

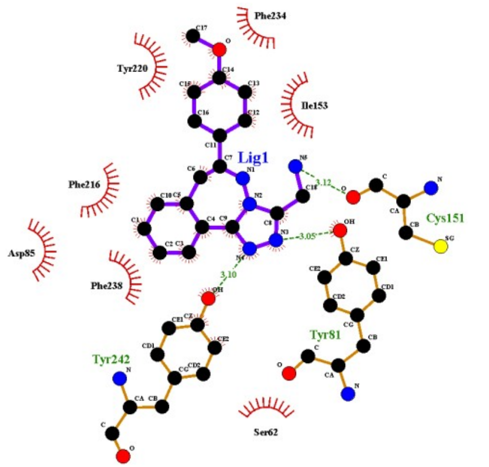

ADRA2A-RD-0014

Type-1 angiotensin II receptor AGTR1

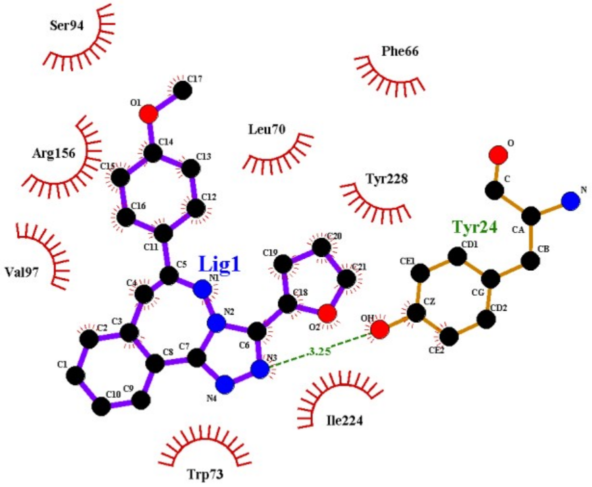

AGTR1-RD-0004

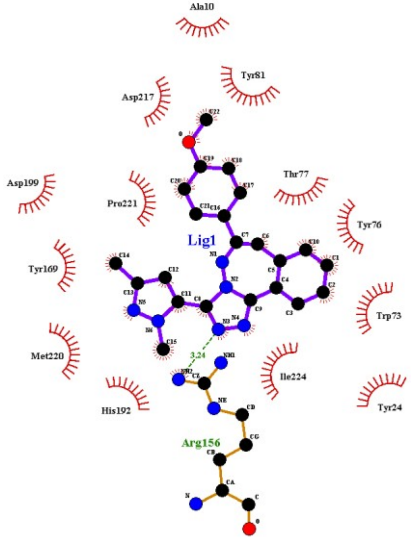

AGTR1-RD-0012

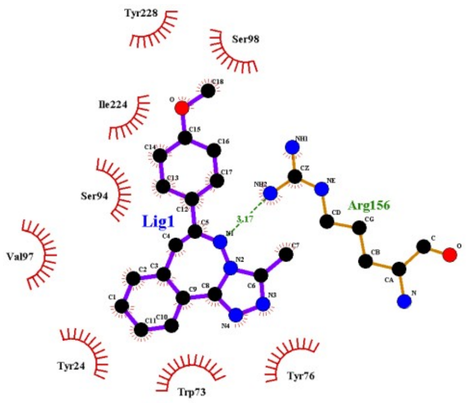

AGTR1-RD-0013

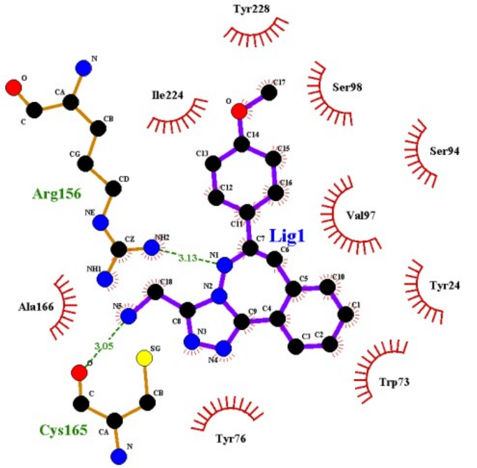

AGTR1-RD-0014

NMDA receptor GluN1/GluN2A/GluN2B GRIN1/GRIN2A/GRIN2B

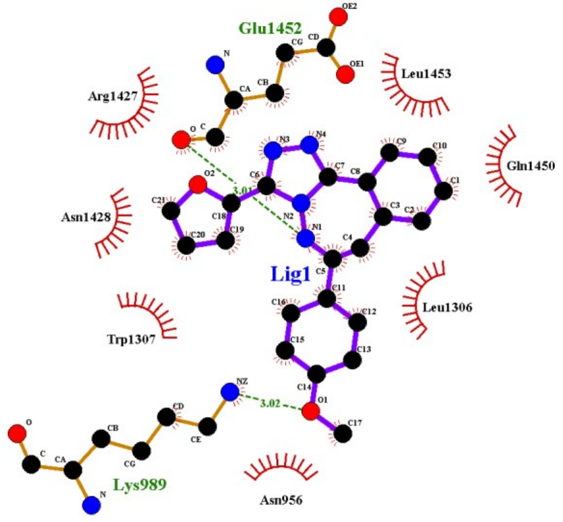

NMDA-Glut-RD-0004

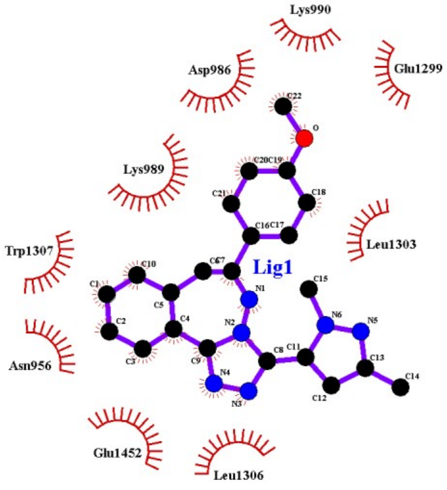

NMDA-Glut-RD-0012

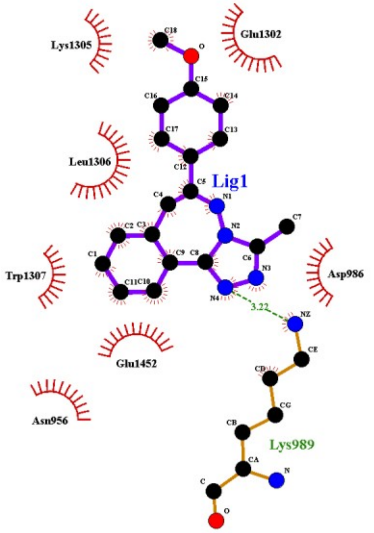

NMDA-Glut-RD-0013

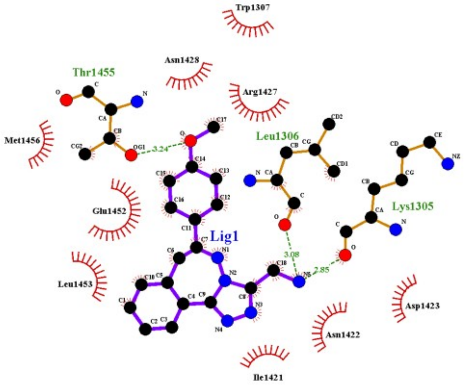

NMDA-Glut-RD-0014
